# The Nutritional Significance of *Ganoderma lucidum* on Human Health: A GRADE‐Assessed Systematic Review and Meta‐Analysis of Clinical Trials

**DOI:** 10.1002/fsn3.70423

**Published:** 2025-06-12

**Authors:** Ali Jafari, Helia Mardani, Zahra Mirzaei Fashtali, Bahareh Arghavan

**Affiliations:** ^1^ Department of Community Nutrition, Faculty of Nutrition Sciences and Food Technology, National Nutrition and Food Technology Research Institute Shahid Beheshti University of Medical Sciences Tehran Iran; ^2^ Systematic Review and Meta‐Analysis Expert Group (SRMEG) Universal Scientific Education and Research Network (USERN) Tehran Iran; ^3^ Department of Nutrition, School of Nutritional Sciences and Dietetics Tehran University of Medical Sciences (TUMS) Tehran Iran; ^4^ Semnan University of Medical Sciences Semnan Iran; ^5^ Department of Basic Medical Sciences, School of Medicine Abadan University of Medical Sciences Abadan Iran

**Keywords:** anthropometric indices, blood pressure, *Ganoderma lucidum*, glycemic indices, lipid profile, liver function

## Abstract

This study aimed to assess the impact of *Ganoderma lucidum* supplementation (200–11,200 mg/day, 1–24 weeks) on health‐related indices in populations including healthy, at‐risk, and chronic disease individuals. A systematic review and meta‐analysis were conducted using data from EMBASE, PubMed, Web of Science, and Scopus up to August 2024. Pooled analysis of 17 randomized controlled trials (RCTs) involving 971 participants revealed that *Ganoderma lucidum* supplementation demonstrated significant reductions in body mass index (BMI) (Weighted Mean Difference [WMD] = −0.43; 95% Confidence Interval [CI]: −0.77, −0.10; *p* = 0.011), creatinine (WMD = −0.14; 95% CI: −0.27, −0.02; *p* = 0.028), glutathione peroxidase (GPx) (WMD = 2.29; 95% CI: 1.67, 2.92; *p* < 0.001), and heart rate (HR) (WMD = −3.92; 95% CI: −7.45, −0.40; *p* = 0.029). No significant effects were observed on body fat, waist circumference, blood pressure, fasting glucose, lipid profile, inflammatory markers, or liver enzymes. Subgroup analyses indicated effects varied by health condition, dosage, duration, and age. Subgroup analyses revealed variations in the effects of *Ganoderma* supplementation based on health conditions, dosage, intervention duration, age at intervention, country, sample size, and publication year. Significant improvements were observed in BMI, TC, creatinine, and MDA in specific subgroups, such as those receiving lower doses (< 1400 mg/day) or those aged < 50 years. The GRADE profile for *Ganoderma* supplementation evaluated the certainty of the outcomes and indicated that the quality of evidence was very low across all outcomes. *Ganoderma lucidum* supplementation may have modest effects on certain health indices, but the evidence is limited by very low quality.

## Graphical abstract

*Ganoderma lucidum* supplementation significantly reduced body mass index, creatinine, glutathione peroxidase, and heart rate. No significant impact was observed on body fat, waist circumference, weight, waist‐to‐hip ratio, diastolic blood pressure, systolic blood pressure, fasting glucose, high‐sensitivity C‐reactive protein, tumor necrosis factor‐alpha, high‐density lipoprotein cholesterol, low‐density lipoprotein cholesterol, total cholesterol, triglycerides, alanine aminotransferase, aspartate aminotransferase, ferric reducing ability of plasma, malondialdehyde, and superoxide dismutase. Subgroup analyses revealed variations in Ganoderma supplementation effects based on health conditions, dosage, intervention duration, intervention age, and country.

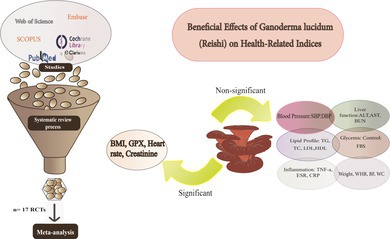

**Abbreviations**
ACC: acetyl‐CoA carboxylase
ACOX1: acyl‐CoA oxidase 1
AFib: atrial fibrillation
ALT: alanine aminotransferase
API: active pharmaceutical ingredient
AST: aspartate aminotransferase
BF: body fat
BMI: body mass index
BP: blood pressure
CHD: coronary heart disease
CI: confidence interval
CMC: Chemistry, Manufacturing, and Controls
CMS: cardiometabolic syndrome
CRP: C‐reactive protein
CVD: cardiovascular disease
DB: double‐blind
DBP: diastolic blood pressure
DNA: deoxyribonucleic acid
FDA: U.S. Food and Drug Administration
FPG: fasting plasma glucose
FRAP: ferric reducing ability of plasma
GPx: glutathione peroxidase
HbA1c: hemoglobin A1c
HDL‐C: high‐density lipoprotein cholesterol
HFD: high‐fat diet
hs‐CRP: high‐sensitivity C‐reactive protein
HTN: hypertension
IL‐6: interleukin‐6
LDL‐C: low‐density lipoprotein cholesterol
MDA: malondialdehyde
MetS: metabolic syndrome
PC: placebo‐controlled
PRISMA: preferred reporting items for systematic reviews and meta‐analyses
PROSPERO: international prospective register of systematic reviews
R: randomized
RA: rheumatoid arthritis
RCTs: randomized controlled trials
SBP: systolic blood pressure
SOD: superoxide dismutase
T2DM: type 2 diabetes mellitus
TC: total cholesterol
TG: triglycerides
TNF‐α: tumor necrosis factor‐alpha
WC: waist circumference
WHO: World Health Organization
WHR: waist‐to‐hip ratio
WMD: weighted wean difference

## 1 Introduction

Cardiovascular disease (CVD) has profound implications for society, the economy, and individual well‐being. According to the World Health Organization (WHO), CVD accounts for approximately 17 million fatalities worldwide annually (Organization WH 2011). Among its manifestations, coronary heart disease (CHD) stands as a primary contributor to worldwide mortality, while stroke remains a leading cause of disability across numerous nations (Organization WH 2011). With the escalating prevalence of diabetes and obesity across both industrialized and developing countries, CVD‐related morbidity and mortality rates are expected to show concurrent increases (Capewell et al. 2010; Hossain et al. 2007; Rahimlou et al. 2022).

The etiology of CVD encompasses numerous factors. Scientists have identified more than 300 variables that may be linked to the development of vascular disease, with their interrelationships potentially leading to complex metabolic consequences. Primary modifiable risk determinants include the use of tobacco products, alcohol intake, hypertension (HTN), elevated lipids, high blood glucose, and excess body weight. Modifiable risk factors, including tobacco use, alcohol consumption, HTN, dyslipidemia, hyperglycemia, and obesity, significantly contribute to CVD burden (Capewell et al. 2010; Hossain et al. 2007; Rahimlou et al. 2022). Recent research has also highlighted emerging biomarkers for their potential in predicting and understanding CVD pathogenesis, including homocysteine levels, inflammatory indicators such as C‐reactive protein (CRP), and coagulation markers like fibrinogen (Pearson et al. 2003).

Herbal compounds have shown promise in managing CVD risk factors, with some demonstrating positive effects (Vahdat et al. 2020; Morshedzadeh et al. 2021; Morvaridi et al. 2020). However, previous trials on *Ganoderma lucidum* have been limited by small sample sizes, inconsistent methodologies, and variable supplement formulations, necessitating a comprehensive synthesis of the evidence.

For more than 2000 years, *Ganoderma lucidum* ( *G. lucidum* ), known traditionally as “lingzhi” or “reishi,” has been valued in Asian medicine and has recently garnered attention in Western medical practice. This fungus occupies a unique position as both a therapeutic and nutritional agent, attributed with properties that may support health maintenance, extend longevity, and address various systemic conditions (Willard 1990). Research interest in *G. lucidum* has grown due to its demonstrated ability to safely influence several CVD risk factors. While traditional preparation methods such as decoctions, teas, and coffee remain available, Western markets increasingly favor standardized extracts in tablet and capsule formations. The organism comprises three main components: the fruiting body (basidiocarp), mycelium (thread‐like structure), and reproductive spores. Its bioactive constituents encompass various compounds including polysaccharides (notably beta‐D‐glucans, heteropolysaccharides, and glycoproteins), triterpenes, germanium, amino acids (both essential and nonessential), sterols, lipids, antioxidants, B‐complex vitamins (B1, B2, and B6), and minerals including iron, calcium, and zinc. Its bioactive compounds, including polysaccharides (e.g., beta‐D‐glucans) and triterpenes, are hypothesized to exert therapeutic effects. Polysaccharides may reduce oxidative stress by enhancing antioxidant enzyme activity, such as glutathione peroxidase (GPx), and modulate inflammation via Toll‐like receptor (TLR) interactions (Huie and Di 2004). Triterpenes may lower blood pressure (BP) by inhibiting angiotensin‐converting enzyme (ACE) and improve lipid profiles through antioxidant upregulation (Huie and Di 2004; McKenna et al. 2012).

These compounds are believed to underpin *G. lucidum'*s therapeutic potential, particularly about cardiovascular and metabolic health. For instance, beta‐D‐glucans, a major polysaccharide class, have been shown to modulate immune responses and reduce systemic inflammation by interacting with TLRs and suppressing pro‐inflammatory cytokines such as tumor necrosis factor‐alpha (TNF‐α) and Interleukin‐6 (IL‐6), which are critical in the pathogenesis of atherosclerosis and insulin resistance (Klupp et al. 2015). Triterpenes, another key constituent, exhibit potential antihypertensive effects through inhibition of ACE activity and reduction of oxidative stress via upregulation of antioxidant enzymes such as superoxide dismutase (SOD) and catalase, thereby contributing to improved lipid profiles and vascular function (Abdullah 2018). Outcomes in this review were selected based on their relevance to cardiovascular and metabolic health, supported by preclinical evidence suggesting *Ganoderma*'s effects on oxidative stress, inflammation, and lipid metabolism (Klupp et al. 2015; Abdullah 2018).

Clinical investigations have revealed *G. lucidum* 's capacity to lower BP, cholesterol, and glucose levels (Gao, Chen, et al. 2004; Gao, Lan, et al. 2004; Kanmatsuse et al. 1985; Jin et al. 1996; Wachtel‐Galor, Tomlinson, et al. 2004). These studies reported no adverse effects on hepatic or renal function markers, and participants experienced no significant adverse reactions. While optimal dosing protocols remain unstandardized, current recommendations typically suggest daily dry extract consumption between 1.5 and 9 g (Chang and Miles 2004; Soo 1996). Although some sources suggest enhanced potency in spore preparations, scientific validation of these claims and their therapeutic implications remains pending.

To address this critical gap in the literature, and given the significance of systematic reviews and meta‐analyses of RCTs in clinical decision‐making, this study was conducted to provide a Grading of Recommendations Assessment, Development and Evaluation (GRADE)‐assessed systematic review and dose–response meta‐analysis of clinical trials. We systematically evaluated RCTs to investigate the associations between factors such as *Ganoderma* dosage and treatment duration and various health parameters. The objective of this research is to systematically evaluate the nutritional and therapeutic effects of *G. lucidum* on human health, with a particular focus on its impact on cardiovascular and metabolic outcomes, through the application of rigorous systematic review and dose–response meta‐analysis methodologies utilizing the GRADE framework. By examining data from RCTs, our findings aim to provide valuable, evidence‐based insights for clinical and public health recommendations, emphasizing *Ganoderma*'s potential role in the prevention and management of cardiovascular and metabolic diseases.

## 2 Methods

### 2.1 Protocol and Registration

This systematic review and meta‐analysis was conducted according to a predefined protocol aligned with the Preferred Reporting Items for Systematic Reviews and Meta‐Analyses (PRISMA) 2020 guidelines to ensure methodological rigor and transparency throughout the process (Moher et al. 2015). The research protocol was registered in the International Prospective Register of Systematic Reviews (PROSPERO), an international database for systematic reviews, under the unique identifier CRD42025632414.

### 2.2 Literature Search and Study Selection

We conducted a comprehensive search across multiple major electronic databases: MEDLINE (via PubMed), Scopus, EMBASE, Cochrane Library, and Web of Science, without restrictions regarding article language or reporting language, up to August 2024. Articles were identified through a manual search of the reference lists of retrieved publications and other pertinent review articles. The review focused on studies that directly reported RCTs examining the effects of supplementation with *G. lucidum* on health‐related outcomes.

Search methodologies were tailored to each individual database, incorporating essential terminology including “*Ganoderma lucidum*,” “Reishi,” and “clinical trial,” in accordance with relevant MeSH terminology and EMTREE keywords (Table S1).

### 2.3 Inclusion/Exclusion Criteria and Data Extraction

Eligible studies were RCTs involving human participants aged ≥ 18 years, comparing *Ganoderma lucidum* supplementation (e.g., extracts, spore powder, capsules, or tablets) with a control group and reporting health‐related outcomes. No minimum intervention duration was set to ensure a comprehensive review. Exclusion criteria included animal/in vitro studies, non‐RCT designs, and studies lacking sufficient data for effect size calculation.

Two independent investigators (A.J. and H.M.) conducted the initial screening of titles and abstracts to determine study relevance. Selected publications subsequently underwent comprehensive full‐text evaluation to confirm their eligibility for inclusion. In cases where consensus could not be reached, a third reviewer (B.A.) was consulted to resolve discrepancies. The research team utilized EndNote version 7.0 for reference organization and elimination of duplicate entries. Data extraction proceeded according to a predefined template that captured key information including study characteristics, participant demographics, intervention specifications, and reported outcomes.

In crossover trials, data were exclusively extracted from the first treatment period to minimize potential carryover effects. When adequate washout periods and adjustments for intra‐subject variability were reported, paired mean differences were utilized. In contrast, studies without such adjustments were analyzed as parallel‐group trials.

### 2.4 Quality Assessment and Evidence Grading

To evaluate methodological rigor, we employed the Cochrane Risk of Bias 2.0 tool (Higgins et al. 2011). Two reviewers (A.J. and Z.M.) independently conducted quality assessments examining multiple methodological domains: randomization procedures, allocation concealment methods, blinding protocols, outcome data completeness, and potential reporting bias. When evaluative differences emerged between reviewers, resolution was achieved through collaborative discussion or arbitration by a third reviewer (B.A.).

The evaluation of evidence certainty followed the GRADE framework methodology, which categorizes evidence quality as high, moderate, low, or very low. This systematic assessment incorporated multiple evaluative criteria, including research design, result consistency, precision of findings, evidence directness, and potential publication bias implications.

### 2.5 Statistical Analysis

Meta‐analytic calculations were performed using Stata version 15.0 (StataCorp, College Station, Texas). The analysis incorporated pre‐ and post‐intervention data, including means, standard deviations, and participant numbers, from eligible RCTs. For studies in which direct statistical measures were unavailable, missing data were estimated using standard formulas or solicited from the authors whenever feasible. Pooled effect sizes were computed using weighted mean differences (WMDs) accompanied by corresponding 95% confidence intervals (CIs) for cross‐study comparisons.

A random‐effects model was applied utilizing the DerSimonian and Laird method to account for variability among studies. The choice of a random‐effects model was based on the anticipated heterogeneity among the included studies, given differences in populations, intervention protocols, dosages, and outcome measurements. This model not only considers within‐study sampling error but also incorporates between‐study variability, resulting in more conservative and generalizable pooled estimates. The presence of substantial clinical and methodological heterogeneity further justified this approach.

Statistical heterogeneity was evaluated using the *I* ^2^ statistic, with thresholds of 25%, 50%, and 75% interpreted as indicating low, moderate, and high heterogeneity, respectively.

For studies in which direct statistical measures were unavailable, missing data were estimated using established formulas recommended in the literature. Specifically, when studies reported standard errors instead of standard deviations (SDs), SD was calculated using the formula: SD = SE × sqrt (*n*), where *n* represents the sample size. In instances where studies provided medians along with ranges or interquartile ranges (IQRs), we applied the methods described by Hozo et al. (Hozo et al. 2005) and Wan et al. (Wan et al. 2014) to estimate the mean and SD. Additionally, for studies reporting only pre‐ and post‐intervention outcomes without the SD of the change, the SD of the change was computed using: (SD = square root [(SD pre‐treatment)^2^ + (SD post‐treatment)^2^ − (2R × SD pre‐treatment × SD post‐treatment)]) with an assumed correlation coefficient *R* of 0.5 in the absence of reported values. These approaches ensure consistency and robustness in our meta‐analytic calculations.

Subgroup analyses were stratified according to health condition (chronic disease conditions including cardiometabolic syndrome (CMS), high‐risk CHD, HTN/dyslipidemia, type 2 diabetes mellitus (T2DM) & metabolic syndrome, asthma, atrial fibrillation (AFib), rheumatoid arthritis (RA), and stable angina pectoris; at‐risk but generally healthy individuals, including those who are overweight, obese, exhibit mild liver dysfunction, have dyslipidemia, and elderly subjects; and healthy conditions encompassing healthy individuals, those in good general health, and subjects free from neurodegenerative disease), Ganoderma dosage (≤ 1400 mg/day vs. > 1400 mg/day), intervention duration (≤ 8 weeks vs. > 8 weeks), age (≤ 50 years vs. > 50 years), geographical region (East Asia, including China, Hong Kong, Taiwan, and Indonesia; the Middle East, including Israel and Iran; and regions encompassing America, Oceania, and Europe, including the USA, Australia, Brazil, and Spain), sample size (≤ 40 vs. > 40 participants), and publication year (≤ 2019 vs. > 2019). Meta‐regression was not conducted due to the limited number of studies per outcome (< 10), as per Cochrane guidelines.

Influence analyses were conducted to assess the effect of each study on the overall findings. The presence of publication bias was examined using Egger's and Begg's tests.

## 3 Results

### 3.1 Study Selection

**FIGURE 1:**
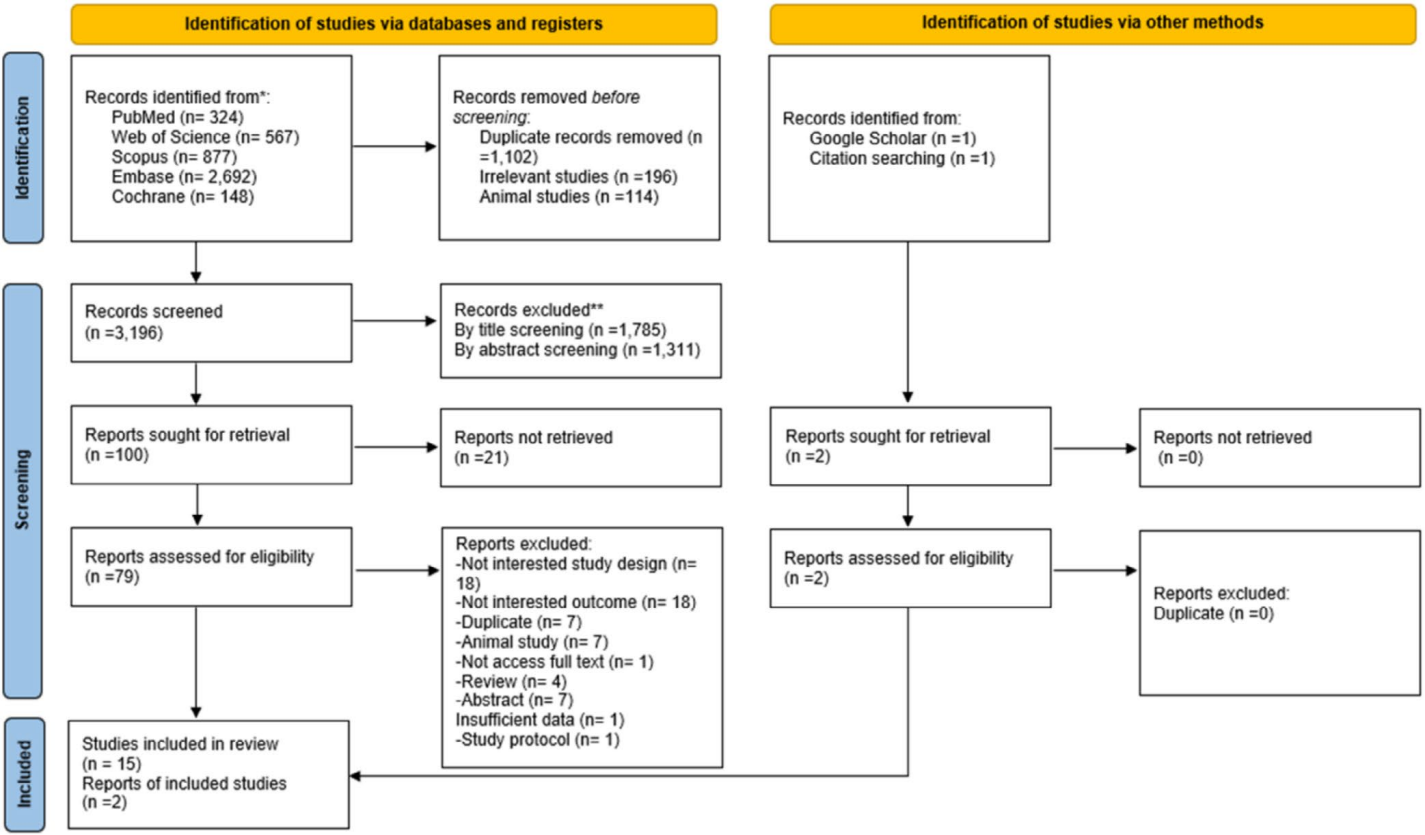
Flowchart of study selection for inclusion trials in the systematic review.

The study selection process is illustrated in Figure 1. A total of 4608 studies were identified through database searches: PubMed (*n* = 324), ISI Web of Science (*n* = 567), Scopus (*n* = 877), Embase (*n* = 2692), and the Cochrane Library (*n* = 148). After the removal of duplicates (*n* = 1102), irrelevant studies (*n* = 196), and animal studies (*n* = 114), 3196 studies were screened based on titles and abstracts. Of these, 3117 were excluded, resulting in 79 full‐text studies undergoing further evaluation. Sixty‐four studies were excluded for not reporting the desired outcomes, as detailed in Table S2. Consequently, 17 studies comprising 971 participants were included in the systematic review and meta‐analysis.

### 3.2 Study Characteristics

The characteristics of the included studies are summarized in Table 1. The WMDs and 95% CIs for changes in body mass index (BMI), body fat (BF), waist circumference (WC), weight, waist‐to‐hip ratio (WHR), diastolic blood pressure (DBP), systolic blood pressure (SBP), fasting glucose, high‐sensitivity C‐reactive protein (hs‐CRP), TNF‐α, high‐density lipoprotein cholesterol (HDL‐C), low‐density lipoprotein cholesterol (LDL‐C), total cholesterol (TC), triglycerides (TG), alanine aminotransferase (ALT), aspartate aminotransferase (AST), creatinine, ferric reducing ability of plasma (FRAP), glutathione peroxidase (GPx), malondialdehyde (MDA), SOD, and heart rate (HR) are demonstrated in Figures S1–S8; funnel plots for these outcomes are presented in Figure S9.

**TABLE 1:** Characteristics of included studies in the meta‐analysis.

Studies year (Ref.)	Country	Study design	Health condition	Sample size (Sex)	Sample size (INT/CON)	Trial duration (Week)	Means age & BMI (INT/CON)	Dose of supplement (mg/d)	Type of supplement (INT/CON)	Outcomes
Wachtel‐Galor, Tomlinson, et al. (2004)	China	PC, DB, cross over	Healthy	36 (NR)	18/18	4	Age: 32 ± 10/32 ± 10 BMI: NR	1440	Capsule: *Ganoderma lucidum* (Lingzhi)/Placebo Ingested with 200–400 mL of warm water	FRAP, TC, TG, HDL‐C, LDL‐C, hs‐CRP, SOD, GPx, MDA, ALT, AST & Creatinine
Wachtel‐Galor, Szeto, et al. (2004)	China	PC, DB, cross over	Healthy	10 (B)	5/5	1	Age: 32.5 ± 10.4/32.5 ± 10.4 BMI: 22.6 ± 2.6	720	Capsule: *Ganoderma lucidum* (Lingzhi)/Placebo	FRAP, TC, TG, HDL‐C, LDL‐C, LDL‐C/HDL‐C, SOD, GPx & MDA
Wen et al. (2005)	China	R, PC, DB	Moderate–severe allergic asthma	91 (B)	45/46	4	Age: 44.6 ± 11.3/45.1 ± 12.0 BMI: NR	3600	Capsule: ASHMI (containing *Ganoderma lucidum*) + Placebo similar in appearance to prednisone/Prednisone + ASHMI placebo	IL‐5, IL‐13 & IFN‐γ
Li et al. (2007)	Hong Kong	R, PC, DB	RA	58 (B)	28/30	24	Age: 50 ± 10/50 ± 13 BMI: NR	11,200	Capsule: *Ganoderma lucidum* (Lingzhi) & San Miao San (SMS) containing G lucidum extract, Rhizoma atractylodis (Cangzhu), Cotex phellodendri (Huangbai), and Radix achyranthes Bidentatae (Niuxi)/Placebo	ESR, CRP & FRAP
Kelly‐Pieper et al. (2009)	USA	R, PC, DB	Asthma	20 (B)	12/8	1	Age: 32 ± 7/31 ± 9 BMI: NR	1200	Capsule: ASHMI [(Ling‐Zhi (*Ganoderma lucidum*), Ku‐Shen ( *Sophora flavescens* ), and Gan‐Cao (Glycyrrhiza uralensis))]/Placebo (cornstarch)	IL‐1ra, IL‐3, IL‐9, IL‐12 (P40), IL‐13, IL‐18, IFN‐γ, ICAM‐1, VCAM‐1, Glucose, Creatinine, ALT & AST
Chu et al. (2012)	Hong Kong	R, PC, DB, Cross Over	HTN and/or dyslipidemia	23 (B)	13/10	12	Age: 56 ± 9.3/53.2 ± 6.7 BMI: 26.1 ± 4.90/26.8 ± 3.9	1440	Capsule: *Ganoderma lucidum* (Lingzhi)/Placebo	HDL‐C, TG, LDL‐C/HDL‐C, TC/HDL‐C, SBP, DBP, WHR, BMI, FPG, Insulin, HOMA‐IR, TC, LDL‐C, Apo B, LDL‐C/Apo B, FRAP, MDA, GPx & SOD
Hu et al. (2014)	Hong Kong	R, PC, DB	Dyslipidemia	40 (B)	20/20	12	Age: 57.5 ± 8.4/54.7 ± 9.1 BMI: 25.9 ± 2.8/27.2 ± 4.7	3440	Capsule: Crataegus‐Based Multiherb Formula (containing *Ganoderma lucidum* (Ling Zhi))/Placebo (starch and artificial food coloring)	Weight, TC, HDL‐C, TG, LDL‐C, Non‐HDL‐C, Fasting glucose, HbA1c, Creatinine, Total protein, Albumin, ALT & ALP
Klupp et al. (2016)	Australia	R, PC, DB	T2DM & MetS	84 (B)	54/30	16	Age: 60.2 ± 10/57.1 ± 8.3 BMI: 34.1 ± 6.8/34.4 ± 7.4	3000	Capsule: *Ganoderma lucidum* with or without Cordyceps sinensis/Placebo (excipient)	HbA1c, FPG, Arterial pressure, TG, HDL‐C, WC, BMI, CRP, TC, LDL‐C, Apo A & Apo B
Chiu et al. (2017)	Taiwan	R, PC, DB, Cross Over	Healthy with mild liver dysfunction	42 (B)	21/21	24	Age: 40–54/40–54 BMI: 23.22 ± 2.93/23.26 ± 2.97	225	Capsule: Triterpenoids and polysaccharide peptides‐enriched *Ganoderma lucidum*/Placebo (90%starch and 10% GL residues)	Weight, BF, BMI, TEAC, TBARS, 8‐OH‐Dg, Thiols, GSH, SOD, CAT, G‐6‐PDH, GPx, GR, ALT & AST
Tsuk et al. (2018)	Israel	R, PC, DB	Healthy	96 (B)	49/47	4	Age: 26.3 ± 3.21/26.3 ± 3.21 BMI: 23.53 ± 2.83/22.65 ± 2.30	1294	Oral liquid and capsule: Liquid (contained Cordyceps sinensis + *Ganoderma lucidum* + shititake + bamboo shoots + honey + potassium sorbet + pure water and capsule (contained *Ganoderma lucidum* + Cordyceps sinensis + soya gel)/Placebo (commercial mushroom soup and glucose capsule)) With Cordyceps sinensis liquid	Weight, BF, BMI, SBP, DBP & HR
Gracián‐Alcaide et al. (2020)	Spain	R, PC, DB	Elderly	60 (B)	30/30	14	Age: 85.9 ± 7.8/82.7 ± 9.2 BMI: 25.4 ± 3.8/26.1 ± 4.6	2000	Sachet: Combination of elderberry ( *Sambucus nigra* L.) and reishi (*Ganoderma lucidum*)/Placebo (maltodextrin)	Weight, SBP & DBP
Rizal et al. (2020)	Indonesia	R, PC	Arterial fibrillation	38 (B)	20/18	12	Age:63.61 ± 8.50/62.50 ± 10.50 BMI: 26.30 ± 5.40/26.40 ± 4.00	750	NR: *Ganoderma lucidum* polysaccharide peptide/Placebo	BMI, SBP, DBP, HR, NO, MDA, hs‐CRP, IL‐1b, IL‐6 & TNF‐α
Babamiri et al. (2022a)	Iran	R, PC, DB	Overweight	69 (B)	36/33	6	Age: 37.25/42.52 ± 10.62 BMI: 26.99 ± 1.02/26.84 ± 1.14	750	Capsule: *Ganoderma lucidum*/Placebo (wheat flour)	Weight, BMI, WC, HC, WHR, MUAC, FBS, TC, LDL‐C, HDL‐C, TG, SBP & DBP
Sargowo et al. (2022)	Indonesia	RCT	Obese	68 (NR)	34/34	12	Age: 58.27 ± 2.5/59.5 ± 2.1 BMI: 31.02 ± 6.2/32.01 ± 7.1	540	Capsule: β‐1,3/1,6‐D‐glucan from mycelia extract (*Ganoderma lucidum*)/Placebo	Creatinine, NO, TNF‐α & MDA
Chen et al. (2023)	Taiwan	R, PC, DB	Healthy	135 (B)	70/65	12	Age: 37.72 ± 7.36/39.11 ± 7.52 BMI: NR	200	Capsule: Reishi β‐glucan/Placebo (dextrose monohydrate)	AST, ALT & Creatinine
Wardhani et al. (2023)	Indonesia	R, DB, controlled trial	Cardiometabolic syndrome	62 (NR)	31/31	8	Age: 58.27 ± 2.50/59.50 ± 2.10 BMI: 31.02 ± 6.20/32.01 ± 11.85	750	Capsule: Dried‐GLPP (*Ganoderma lucidum* polysaccharide peptide)/Placebo	TNF‐α, hs‐CRP, MDA, SOD & Creatinine
Iser‐Bem et al. (2024)	Brazil	PC, DB	Free from neurodegenerative disease	39 (F)	23/16	8	Age: 68 ± 5.8/66.4 ± 5.9 BMI: 26.4 ± 5.5/26.5 ± 4.9	2000	Capsule: *Ganoderma lucidum*/Placebo (corn starch)	Weigth, BMI, BF, Lean mass, WC, HC & WHR

Abbreviations: %BF, Body Fat Percentage; 8‐OH‐dG, 8‐Hydroxyguanosine; ALP, Alkaline Phosphatase; ALT, Alanine Aminotransferase; Apo A‐1, Apolipoprotein A‐I; Apo B, Apolipoprotein B; AST, Aspartate Aminotransferase; B, Both; BMI, Body Mass Index; BUN, Blood Urea Nitrogen; CAT, Catalase; CRP, C‐reactive protein; DB, Double Blind; DBP, Diastolic Blood Pressure; ESR, Erythrocyte Sedimentation Rate; F, Female; FBS, Fasting blood sugar; FPG, Fasting Plasma Glucose; FRAP, Ferric reducing ability of plasma; G‐6‐PDH, Glucose‐6‐phosphate dehydrogenase; GLPP, *Ganoderma lucidum* Polysaccharide Peptide; GPx, Glutathione Peroxidase; GR, Glutathione reductase; GSH, Total Glutathione; HbA1c, Hemoglobin A1c; HC, Hip Circumference; HDL‐C, High‐density Lipoprotein Cholesterol; HOMA‐IR, Homeostatic Model Assessment for Insulin Resistance; HR, Heart Rate; hs‐CRP, high‐sensitivity C‐reactive Protein; HTN, Hypertension; ICAM‐1, Intercellular adhesion molecule‐1; IFN‐γ, Interferon‐Gamma; IL‐13, Interleukin‐13; IL‐18, Interleukin‐18; IL‐1b, Interleukin‐1 Beta; IL‐1ra, Interleukin‐1 receptor antagonist; IL‐3, Interleukin‐3; IL‐5, Interleukin‐5; IL‐6, Interleukin‐6; IL‐9, Interleukin‐9; LDL‐C, Low‐density Lipoprotein Cholesterol; M, Male; MDA, Malondialdehyde; MetS, Metabolic syndrome; MUAC, Mid‐upper arm circumference; NO, Nitric Oxide; NR, Not reported; PC, Placebo‐Controlled; R, Randomized; RA, Rheumatoid arthritis; Ref, Reference; SBP, Systolic Blood Pressure; SOD, Superoxide Dismutase activity; T2DM, Type 2 Diabetes Mellitus; TBARS, Thiobarbituric Acid Reactive Substance; TC, Total Cholesterol; TEAC, Trolox‐equivalent antioxidant capacity; TG, Triglycerides; TNF‐α, Tumor Necrosis Factor‐alpha; VCAM‐1, Vascular cell adhesion molecule 1; WC, Waist Circumference; WHR, Waist‐to‐Hip Ratio.

The studies were published between 2004 and 2024 and were conducted in various countries, including Australia (Klupp et al. 2016), Brazil (Iser‐Bem et al. 2024), China (Wachtel‐Galor, Tomlinson, et al. 2004; Wachtel‐Galor, Szeto, et al. 2004; Wen et al. 2005), Hong Kong (Li et al. 2007; Chu et al. 2012; Hu et al. 2014), Indonesia (Rizal et al. 2020; Sargowo et al. 2022; Wardhani et al. 2023), Iran (Babamiri et al. 2022a), Israel (Tsuk et al. 2018), Spain (Gracián‐Alcaide et al. 2020), Taiwan (Chiu et al. 2017; Chen et al. 2023), and the USA (Kelly‐Pieper et al. 2009). Participant ages in the intervention groups ranged from 26.3 to 85.9 years, with Ganoderma doses varying from 200 to 11,200 mg/day over intervention durations of 1–24 weeks. Sample sizes ranged from 10 to 135 participants. Participants presented with conditions such as moderate to severe allergic asthma (Wen et al. 2005), RA (Li et al. 2007) asthma (Kelly‐Pieper et al. 2009), HTN and/or dyslipidemia (Chu et al. 2012; Hu et al. 2014), T2DM and metabolic syndrome (MetS) (Klupp et al. 2016), mild liver dysfunction (Chiu et al. 2017), elderly status (Gracián‐Alcaide et al. 2020), AFib (Rizal et al. 2020), overweight (Babamiri et al. 2022a), obesity (Sargowo et al. 2022), CMS (Wardhani et al. 2023), absence of neurodegenerative disease (Iser‐Bem et al. 2024) and general health or non‐specific conditions (Wachtel‐Galor, Tomlinson, et al. 2004; Wachtel‐Galor, Szeto, et al. 2004; Tsuk et al. 2018; Chen et al. 2023).

The sample sizes for the intervention and control groups across various outcomes were as follows: BMI: *n* = 392 (intervention: 216, control: 176); BF: *n* = 178 (intervention: 93, control: 85); WC: *n* = 192 (intervention: 113, control: 79); weight: *n* = 347 (intervention: 179, control: 168); WHR: *n* = 131 (intervention: 72, control: 59); DBP: *n* = 287 (intervention: 148, control: 139); SBP: *n* = 287 (intervention: 148, control: 139); fasting glucose: *n* = 236 (intervention: 135, control: 101); hs‐CRP: *n* = 136 (intervention: 69, control: 67); TNF‐α: *n* = 168 (intervention: 85, control: 83); HDL‐C: *n* = 262 (intervention: 146, control: 116); LDL‐C: *n* = 262 (intervention: 146, control: 116); TC: *n* = 262 (intervention: 146, control: 116); TG: *n* = 262 (intervention: 146, control: 116); ALT: *n* = 273 (intervention: 141, control: 132); AST: *n* = 233 (intervention: 121, control: 112); creatinine: *n* = 361 (intervention: 185, control: 176); FRAP: *n* = 127 (intervention: 64, control: 63); GPx: *n* = 88 (intervention: 44, control: 44); MDA: *n* = 237 (intervention: 121, control: 116); SOD: *n* = 163 (intervention: 83, control: 80); HR: *n* = 135 (intervention: 69, control: 66).

### 3.3 Qualitative Data Assessment

Utilizing the Cochrane Risk of Bias Assessment tool, none of the studies were rated as good quality, one was rated as fair (Hu et al. 2014), and the remaining studies were classified as poor (Klupp et al. 2016; Iser‐Bem et al. 2024; Wachtel‐Galor, Tomlinson, et al. 2004; Wachtel‐Galor, Szeto, et al. 2004; Wen et al. 2005; Li et al. 2007; Chu et al. 2012; Rizal et al. 2020; Sargowo et al. 2022; Wardhani et al. 2023; Babamiri et al. 2022a; Tsuk et al. 2018; Gracián‐Alcaide et al. 2020; Chiu et al. 2017; Chen et al. 2023; Kelly‐Pieper et al. 2009) (Table 2).

**TABLE 2:** Quality of included studies in the meta‐analysis.

Study, year (Ref.)	Random sequence generation	Allocation concealment	Blinding of participants & personnel	Blinding of outcome assessment	Incomplete outcome data	Selective outcome reporting	Other sources of bias	Overall quality
Wachtel‐Galor, Tomlinson, et al. (2004)	H	H	U	H	L	L	L	Poor
Wachtel‐Galor, Tomlinson, et al. (2004)	H	H	L	H	H	L	L	Poor
Wen et al. (2005)	U	H	L	H	L	L	L	Poor
Li et al. (2007)	L	L	L	H	H	L	L	Poor
Kelly‐Pieper et al. (2009)	U	H	L	H	L	L	L	Poor
Chu et al. (2012)	U	H	U	H	H	L	L	Poor
Hu et al. (2014)	L	H	L	L	L	L	L	Fair
Klupp et al. (2016)	L	H	L	L	H	L	L	Poor
Chiu et al. (2017)	U	H	L	H	L	L	L	Poor
Tsuk et al. (2018)	U	H	H	H	L	L	L	Poor
Gracián‐Alcaide et al. (2020)	U	H	L	H	H	L	L	Poor
Rizal et al. (2020)	L	H	H	H	U	L	L	Poor
Babamiri et al. (2022a)	U	H	U	H	L	L	L	Poor
Sargowo et al. (2022)	L	H	H	H	L	L	L	Poor
Chen et al. (2023)	L	H	U	H	H	L	L	Poor
Wardhani et al. (2023)	U	H	U	H	U	L	L	Poor
Iser‐Bem et al. (2024)	H	H	U	H	H	L	L	Poor

Abbreviations: H, high risk of bias; L, low risk of bias; U, unclear risk of bias.

### 3.4 Effects of *Ganoderma* Supplementation on Anthropometric Indices

*Ganoderma* supplementation demonstrates significant effects on BMI (WMD = −0.43, 95% CI: −0.77 to −0.10; *p* = 0.011; *I* ^2^ = 40.8%, *p* = 0.106) (Figure S1). However, *Ganoderma* supplementation did not show significant effects on BF (WMD = −0.42, 95% CI: −1.55 to 0.71; *p* = 0.467; *I* ^2^ = 0.0%, *p* = 0.647), WC (WMD = −0.45, 95% CI: −1.02 to 0.12; *p* = 0.123; *I* ^2^ = 0.0%, *p* = 0.753), weight (WMD = −0.15, 95% CI: −1.85 to 1.55; *p* = 0.865; *I* ^2^ = 0.0%, *p* = 0.725), or WHR (WMD = 0.00, 95% CI: −0.01 to 0.01; *p* = 0.582; *I* ^2^ = 18.5%, *p* = 0.293).

Sensitivity analyses indicated that excluding the study by Babamiri et al. (Babamiri et al. 2022a) (WMD: ‐0.41, 95% CI: −0.86, 0.29) significantly affected BMI. Egger's test did not indicate significant publication bias for BMI (*p* = 0.774), BF (*p* = 0.202), WC (*p* = 0.179), weight (*p* = 0.678), and WHR (*p* = 0.500).

### 3.5 Effects of *Ganoderma* Supplementation on Blood Pressure

*Ganoderma* supplementation did not demonstrate significant effects on DBP (WMD = −0.72, 95% CI: −3.43 to 1.99; *p* = 0.601; *I* ^2^ = 74.0%, *p* = 0.002) (Figure S2) and SBP (WMD = −1.50, 95% CI: −4.25 to 1.26; *p* = 0.286; *I* ^2^ = 52.4%, *p* = 0.062).

Sensitivity analyses showed consistent results even after excluding individual studies. Egger's test indicated no significant publication bias for DBP (*p* = 0.757) or SBP (*p* = 0.483).

### 3.6 Effects of *Ganoderma* Supplementation on Glycemic Profile

Our meta‐analysis did not show any significant changes in fasting glucose (WMD = −0.03, 95% CI: −0.13 to 0.07; *p* = 0.555; *I* ^2^ = 0.0%, *p* = 0.952) (Figure S3).

Sensitivity analyses showed no alteration in this group's parameter, and there was also no significant publication bias for fasting glucose (*p* = 0.785) based on Egger's test.

### 3.7 Effects of Ganoderma Supplementation on Inflammatory Markers

*Ganoderma* supplementation did not demonstrate significant effects on hs‐CRP (WMD = −0.28, 95% CI: −0.91 to 0.35; *p* = 0.388; *I* ^2^ = 91.9%, *p* < 0.001) (Figure S4), and TNF‐α (WMD = −7.70, 95% CI: −27.84 to 12.43; *p* = 0.453; *I* ^2^ = 89.9%, *p* < 0.001).

Sensitivity analyses demonstrated stability in this group's parameters. Egger's test indicated no significant publication bias for TNF‐α (*p* = 0.725) or hs‐CRP (*p* = 0.682).

### 3.8 Effects of Ganoderma Supplementation on Lipid Profile

The findings from our analysis indicate that *Ganoderma* supplementation does not yield significant benefits on HDL‐C (WMD = 0.02, 95% CI: −0.04 to 0.08; *p* = 0.467; *I* ^2^ = 0.0%, *p* = 0.480) (Figure S5), LDL‐C (WMD = −0.13, 95% CI: −0.34 to 0.08; *p* = 0.225; *I* ^2^ = 54.9%, *p* = 0.050), TC (WMD = −0.18, 95% CI: −0.39 to 0.02; *p* = 0.083; *I* ^2^ = 32.8%, *p* = 0.190), and TG (WMD = −0.12, 95% CI: −0.30 to 0.06; *p* = 0.197; *I* ^2^ = 61.2%, *p* = 0.024).

Sensitivity analyses reveal that the exclusion of the study by Klupp et al. (2016) resulted in a notable change in the overall results concerning TC levels (WMD: −0.27, 95% CI: −0.44 to −0.10). Additionally, the removal of the study by Hu et al. (2014) altered the overall effect on TG (WMD: ‐0.16, 95% CI: −0.31 to −0.01). Furthermore, Egger's test did not provide evidence of publication bias in the lipid profile parameters.

### 3.9 Effects of Ganoderma Supplementation on Liver Function

Ganoderma supplementation demonstrates significant effects on creatinine (WMD = −0.14, 95% CI: −0.27 to −0.02; *p* = 0.028; *I* ^2^ = 80.0%, *p* < 0.001) (Figure S6). However, Ganoderma supplementation did not demonstrate significant effects on ALT (WMD = −2.06, 95% CI: −7.64 to 3.52; *p* = 0.470: *I* ^2^ = 95.1%, *p* < 0.001) and AST (WMD = −1.52, 95% CI: −5.09 to 2.04; *p* = 0.402: *I* ^2^ = 85.6%, *p* < 0.001).

In contrast to other parameters within this category, sensitivity analysis indicated that the removal of studies by Wardhani et al. (2023) (WMD: −0.09, 95% CI: −0.22 to 0.30), Chen et al. (Chen et al. 2023) (WMD: −0.17, 95% CI: −0.39 to 0.05), and Sargowo et al. (Sargowo et al. 2022) (WMD: −0.09, 95% CI: −0.20 to 0.03) influenced the overall results concerning creatinine. Egger's test did not reveal significant publication bias for ALT (*p* = 0.912) and AST (*p* = 0.671).

### 3.10 Effects of Ganoderma Supplementation on Oxidative Stress Parameters

The trial demonstrated a significant effect on GPx (WMD = 2.29, 95% CI: 1.67–2.92; *p* < 0.001; *I* ^2^ = 0.0%, *p* = 0.986) (Figure S7). However, no significant changes were observed in FRAP (WMD = −8.60, 95% CI: −72.07 to 54.87; *p* = 0.791; *I* ^2^ = 47.7%, *p* = 0.125), MDA (WMD = −0.07, 95% CI: −0.14 to 0.01; *p* = 0.071; *I* ^2^ = 96.0%, *p* < 0.001), or SOD (WMD = 76.86, 95% CI: −21.51 to 175.23; *p* = 0.126; *I* ^2^ = 94.3%, *p* < 0.001).

Sensitivity analyses revealed that excluding the study by Chiu et al. (2017) altered the results for GPx (WMD = 2.40, 95% CI: −0.80 to 5.61). Excluding the studies by Wachtel‐Galor, Tomlinson, et al. (2004) (WMD = −0.10, 95% CI: −0.19 to −0.01) and Wachtel‐Galor, Szeto, et al. (2004) (WMD = −0.09, 95% CI: −0.17 to −0.01) affected the overall results for MDA. The study by Chiu et al. (2017) also influenced the sensitivity analysis for SOD (WMD = 10.67, 95% CI: 5.61 to 15.72). No publication bias was detected for oxidative stress parameters.

### 3.11 Effects of Ganoderma Supplementation on Heart Rate

The pooled results from studies investigating the effects of Ganoderma on HR showed a significant decrease (WMD = −3.92, 95% CI: −7.45 to −0.40; *p* = 0.029; *I* ^2^ = 53.5%, *p* = 0.116) (Figure S8).

Sensitivity analyses indicated that excluding the studies by Tsuk et al. (2018) (WMD = −2.07, 95% CI: −9.73 to 1.04) and Rizal et al. (Rizal et al. 2020) (WMD = −4.34, 95% CI: −4.93 to 0.79) altered the pooled results for HR. No publication bias was detected for HR, as confirmed by Egger's test.

### 3.12 Subgroup Analysis

Subgroup analyses based on health conditions (chronic disease vs. at‐risk but generally healthy individuals vs. healthy populations), *Ganoderma* dosage (< 1400 mg/day vs. ≥ 1400 mg/day), intervention duration (≤ 8 weeks vs. > 8 weeks), participant age (< 50 years vs. ≥ 50 years), geographical region (East Asia vs. Middle East vs. America, Oceania, and Europe), sample size (< 40 participants vs. ≥ 40 participants), and publication year (≤ 2019 vs. > 2019) are summarized in Table 3.

**TABLE 3:** Description of the analysis and subgroup results of Ganoderma supplementation on cardiovascular disease risk factors.

	Studies N	Participant N	WMD (95% CI)	*p*	Heterogeneity			
					*p* heterogeneity	*I* ^2^	τ^2^	*p* between subgroups
Analysis and subgroup results of Ganoderma supplementation on BMI								
Overall effect	8	392	−0.43 (−0.77, −0.10)	**0.011**	0.106	40.8%	0.077	
Health condition								
Chronic disease	3	145	−0.13 (−0.43, 0.17)	0.405	0.760	0.0%	NA	0.009
At‐risk but generally healthy individuals	2	111	−0.51 (−0.84, −0.18)	**0.003**	0.418	0.0%	NA	
Healthy	3	136	−1.19 (−1.83, −0.55)	**< 0.001**	0.546	0.0%	NA	
Ganoderma dosage								
1400 <	5	246	−0.58 (−1.07, −0.09)	**0.020**	0.051	57.6%	NA	0.197
1400 ≤	3	146	−0.19 (−0.53, 0.16)	0.297	0.925	0.0%	NA	
Intervention duration								
8 ≥	4	187	−0.82 (−1.30, −0.34)	**0.001**	0.241	28.5%	NA	0.015
8 <	4	205	−0.12 (−0.41, 0.17)	0.410	0.904	0.0%	NA	
Intervention age								
50 >	4	184	−0.77 (−1.28, −0.27)	**0.003**	0.171	40.1%	NA	0.032
50 ≤	4	208	−0.13 (−0.43, 0.17)	0.402	0.908	0.0%	NA	
Geographical region								
East Asia	3	103	−0.13 (−0.42, 0.17)	0.389	0.792	0.0%	NA	0.057
Middle East	3	166	−0.91 (−1.48, −0.33)	**0.002**	0.145	48.3%	NA	
America, Oceania and Europe	2	123	0.05 (−1.38, 1.49)	0.942	0.837	0.0%	NA	
Sample size								
40 >	3	100	−0.14 (−0.44, 0.17)	0.380	0.801	0.0%	NA	0.041
40 ≤	5	292	−0.71 (−1.18, −0.25)	**0.003**	0.222	30.0%	NA	
Publication year								
2019 ≥	5	246	−0.60 (−1.21, 0.02)	0.058	0.064	54.9%	NA	0.476
2019 <	3	146	−0.32 (−0.76, 0.11)	0.147	0.228	32.3%	NA	0.476
Analysis and subgroup results of Ganoderma supplementation on BF								
Overall effect	4	178	−0.42 (−1.55, 0.71)	0.467	0.647	0.0%	0.000	
Analysis and subgroup results of Ganoderma supplementation on WC								
Overall effect	3	192	−0.45 (−1.02, 0.12)	0.123	0.753	0.0%	0.000	
Analysis and subgroup results of Ganoderma supplementation on weight								
Overall effect	7	347	−0.15 (−1.85, 1.55)	0.865	0.725	0.0%	0.000	
Health condition								
Chronic disease	1	40	3.00 (−3.21, 9.21)	0.344	—	—	NA	0.560
At‐risk but generally healthy individuals	3	171	−0.66 (−3.09, 1.78)	0.596	0.348	5.2%	NA	
Healthy	3	136	−0.09 (−2.74, 2.56)	0.948	0.833	0.0%	NA	
Ganoderma dosage								
1400 <	4	208	−0.56 (−2.68, 1.56)	0.607	0.559	0.0%	NA	0.529
1400 ≤	3	139	0.58 (−2.25, 3.42)	0.687	0.555	0.0%	NA	
Intervention duration								
8 ≥	4	205	−0.97 (−3.22, 1.28)	0.396	0.591	0.0%	NA	0.273
8 <	3	142	0.95 (−1.64, 3.54)	0.474	0.768	0.0%	NA	
Intervention age								
50 >	4	208	−0.56 (−2.68, 1.56)	0.607	0.559	0.0%	NA	0.529
50 ≤	3	139	0.58 (−2.25, 3.42)	0.687	0.555	0.0%	NA	
Geographical region								
East Asia	2	82	1.14 (−2.33, 4.61)	0.518	0.480	0.0%	NA	0.654
Middle East	3	166	−0.85 (−3.31, 1.61)	0.498	0.396	0.0%	NA	
America, Oceania and Europe	2	99	−0.06 (−3.24, 3.14)	0.973	0.507	0.0%	NA	
Sample size								
40 >	1	39	−1.60 (−7.17, 3.97)	0.573	—	—	NA	0.591
40 ≤	6	308	0.00 (−1.78, 1.78)	0.999	0.646	0.0%	NA	
Publication year								
2019 ≥	4	179	0.69 (−1.58, 2.96)	0.551	0.893	0.0%	NA	0.276
2019 <	3	168	−1.21 (−3.76, 1.35)	0.354	0.398	0.0%	NA	0.276
Analysis and subgroup results of Ganoderma supplementation on WHR								
Overall effect	3	131	0.00 (−0.00, 0.01)	0.582	0.293	18.5%	0.000	
Analysis and subgroup results of Ganoderma supplementation on DBP								
Overall effect	6	287	−0.72 (−3.43, 1.99)	0.601	0.002	74.0%	8.003	
Health condition								
Chronic disease	2	61	0.67 (−8.34, 9.67)	0.884	0.025	80.0%	NA	0.695
At‐risk but generally healthy individuals	2	129	−2.59 (−7.71, 2.54)	0.323	0.020	81.7%	NA	
Healthy	2	97	−0.23 (−2.85, 2.38)	0.861	0.169	47.2%	NA	
Ganoderma dosage								
1400 <	4	204	−0.30 (−1.96, 1.36)	0.725	0.345	9.7%	NA	0.996
1400 ≤	2	83	−0.28 (−10.07, 9.52)	0.956	< 0.001	93.5%	NA	
Intervention duration								
8 ≥	3	166	−0.02 (−1.60, 1.56)	0.977	0.388	0.0%	NA	0.698
8 <	3	121	−1.48 (−8.64, 5.67)	0.686	< 0.001	87.5%	NA	
Intervention age								
50 >	3	166	−0.02 (−1.60, 1.56)	0.977	0.388	0.0%	NA	0.698
50 ≤	3	121	−1.48 (−8.64, 5.69)	0.686	< 0.001	87.5%	NA	
Geographical region								
East Asia	2	61	0.67 (−8.34, 9.67)	0.884	0.025	80.0%	NA	0.013
Middle East	3	166	−0.02 (−1.60, 1.56)	0.977	0.388	0.0%	NA	
America, Oceania and Europe	1	60	−5.20 (−8.30, −2.10)	**0.001**	—	—	NA	
Sample size								
40 >	2	61	0.67 (−8.34, 9.67)	0.884	0.025	80.0%	NA	0.664
40 ≤	4	226	−1.42 (−4.09, 1.26)	0.299	0.015	71.1%	NA	
Publication year								
2019 ≥	3	120	1.10 (−2.12, 4.32)	0.504	0.035	70.1%	NA	0.117
2019 <	3	167	−2.95 (−6.85, 0.95)	0.139	0.058	64.9%	NA	
Analysis and subgroup results of Ganoderma supplementation on SBP								
Overall effect	6	287	−1.50 (−4.25, 1.26)	0.286	0.062	52.4%	5.748	
Health condition								
Chronic disease	2	61	−4.00 (−18.67, 10.67)	0.593	0.019	81.8%	NA	0.797
At‐risk but generally healthy individuals	2	129	−2.19 (−7.71, 3.34)	0.438	0.034	77.8%	NA	
Healthy	2	97	−0.53 (−3.20, 2.15)	0.699	0.944	0.0%	NA	
Ganoderma dosage								
1400 <	4	204	−0.95 (−3.83, 1.93)	0.519	0.166	40.9%	NA	0.878
1400 ≤	2	83	−1.60 (−9.47, 6.26)	0.690	0.050	74.0%	NA	
Intervention duration								
8 ≥	3	166	−0.12 (−2.22, 1.98)	0.912	0.887	0.0%	NA	0.291
8 <	3	121	−4.06 (−11.07, 2.95)	0.256	0.042	68.5%	NA	
Intervention age								
50 >	3	166	−0.12 (−2.22, 1.98)	0.912	0.887	0.0%	NA	0.291
50 ≤	3	121	−4.06 (−11.07, 2.95)	0.256	0.042	68.5%	NA	
Geographical region								
East Asia	2	61	−4.00 (−18.67, 10.67)	0.593	0.019	81.8%	NA	0.087
Middle East	3	166	−0.12 (−2.22, 1.98)	0.912	0.887	0.0%	NA	
America, Oceania and Europe	1	60	−5.10 (−9.06, −1.14)	**0.012**	—	—	NA	
Sample size								
40 >	2	61	−4.00 (−18.67,10.67)	0.593	0.019	81.8%	NA	0.721
40 ≤	4	226	−1.29 (−3.71, 1.12)	0.294	0.173	39.9%	NA	
Publication year								
2019 ≥	3	120	−0.09 (−2.59, 2.42)	0.947	0.656	0.0%	NA	0.216
2019 <	3	167	−4.06 (−9.83, 1.71)	0.168	0.019	74.9%	NA	
Analysis and subgroup results of Ganoderma supplementation on Fasting glucose								
Overall effect	5	236	−0.03 (−0.13, 0.07)	0.555	0.952	0.0%	0.000	
Health condition								
Chronic disease	4	167	−0.03 (−0.24, 0.19)	0.801	0.874	0.0%	NA	0.984
At‐risk but generally healthy individuals	1	69	−0.03 (−0.14, 0.08)	0.594	—	—	NA	
Healthy	—	—	—	—	—	—	NA	
Ganoderma dosage								
1400 <	2	89	−0.02 (−0.13, 0.09)	0.687	0.565	0.0%	NA	0.725
1400 ≤	3	147	−0.07 (−0.32, 0.18)	0.578	0.886	0.0%	NA	
Intervention duration								
8 ≥	2	89	−0.02 (−0.13, 0.09)	0.687	0.565	0.0%	NA	0.725
8 <	3	147	−0.07 (−0.32, 0.18)	0.578	0.886	0.0%	NA	
Intervention age								
50 >	2	89	−0.02 (−0.13, 0.09)	0.687	0.565	0.0%	NA	0.725
50 ≤	3	147	−0.07 (−0.32, 0.18)	0.578	0.886	0.0%	NA	
Geographical region								
East Asia	2	63	−0.07 (−0.33, 0.19)	0.591	0.623	0.0%	NA	0.845
Middle East	1	69	−0.03 (−0.14, 0.08)	0.594	—	—	NA	
America, Oceania and Europe	2	104	0.06 (−0.31, 0.44)	0.740	0.728	0.0%	NA	
Sample size								
40 >	2	43	−0.01 (−0.24, 0.23)	0.959	0.564	0.0%	NA	0.827
40 ≤	3	193	−0.04 (−0.14, 0.07)	0.531	0.853	0.0%	NA	
Publication year								
2019 ≥	4	167	−0.03 (−0.24, 0.19)	0.801	0.874	0.0%	NA	0.984
2019 <	1	69	−0.03 (−0.14, 0.08)	0.594	—	—	NA	
Analysis and subgroup results of Ganoderma supplementation on hs‐CRP								
Overall effect	3	136	−0.28 (−0.91, 0.35)	0.388	< 0.001	91.9%	0.279	
Analysis and subgroup results of Ganoderma supplementation on TNF‐alpha								
Overall effect	3	168	−7.70 (−27.84, 12.43)	0.453	< 0.001	89.9%		
Analysis and subgroup results of Ganoderma supplementation on HDL‐C								
Overall effect	6	262	0.02 (−0.04, 0.08)	0.467	0.480	0.0%	0.000	
Health condition								
Chronic disease	3	147	0.02 (−0.08, 0.11)	0.723	0.191	39.5%	NA	0.611
At‐risk but generally healthy individuals	1	69	0.08 (−0.05, 0.21)	0.234	—	—	NA	
Healthy	2	46	−0.01 (−0.14, 0.12)	0.882	0.704	0.0%	NA	
Ganoderma dosage								
1400 <	2	79	0.06 (−0.05, 0.17)	0.271	0.622	0.0%	NA	0.414
1400 ≤	4	183	0.01 (−0.06, 0.08)	0.842	0.317	15.0%	NA	
Intervention duration								
8 ≥	3	115	0.033 (−0.06, 0.13)	0.472	0.586	0.0%	NA	0.805
8 <	3	147	0.02 (−0.08, 0.11)	0.723	0.191	39.5%	NA	
Intervention age								
50 >	3	115	0.03 (−0.06, 0.13)	0.472	0.586	0.0%	NA	0.805
50 ≤	3	147	0.02 (−0.08, 0.11)	0.723	0.191	39.5%	NA	
Geographical region								
East Asia	4	109	0.01 (−0.07, 0.10)	0.753	0.321	14.2%	NA	0.608
Middle East	1	69	0.08 (−0.05, 0.21)	0.234	—	—	NA	
America, Oceania and Europe	1	84	0.00 (−0.09, 0.09)	1.000	—	—	NA	
Sample size								
40 >	3	69	0.05 (−0.05, 0.15)	0.349	0.360	2.2%	NA	0.519
40 ≤	3	193	0.01 (−0.06, 0.08)	0.819	0.363	1.2%	NA	
Publication year								
2019 ≥	5	69	0.01 (−0.05, 0.07)	0.802	0.471	0.0%	NA	0.329
2019 <	1	193	0.08 (−0.05, 0.21)	0.234	—	—	NA	
Analysis and subgroup results of Ganoderma supplementation on LDL‐C								
Overall effect	6	262	−0.13 (−0.34, 0.08)	0.225	0.050	54.9%	0.035	
Health condition								
Chronic disease	3	147	0.03 (−0.33, 0.28)	0.869	0.146	48.1%	NA	0.145
At‐risk but generally healthy individuals	1	69	−0.03 (−0.19, 0.13)	0.712	—	—	NA	
Healthy	2	46	−0.46 (−0.88, −0.05)	**0.027**	0.236	28.8%	NA	
Ganoderma dosage								
1400 <	2	79	−0.05 (−0.20, 0.10)	0.533	0.445	0.0%	NA	0.522
1400 ≤	4	183	−0.18 (−0.55, 0.19)	0.341	0.017	70.7%	NA	
Intervention duration								
8 ≥	3	115	−0.27 (−0.66, 0.12)	0.181	0.038	69.4%	NA	0.339
8 <	3	147	−0.03 (−0.33, 0.28)	0.869	0.146	48.1%	NA	
Intervention age								
50 >	3	115	−0.27 (−0.66, 0.12)	0.181	0.038	69.4%	NA	0.339
50 ≤	3	147	−0.03 (−0.33, 0.28)	0.869	0.146	48.1%	NA	
Geographical region								
East Asia	4	109	−0.30 (−0.56, −0.05)	**0.018**	0.285	20.8%	NA	0.040
Middle East	1	69	−0.03 (−0.19, 0.13)	0.712	—	—	NA	
America, Oceania and Europe	1	84	0.20 (−0.11, 0.51)	0.203	—	—	NA	
Sample size								
40 >	3	69	−0.31 (−0.68, 0.06)	0.105	0.151	47.2%	NA	0.194
40 ≤	3	193	−0.02 (−0.24, 0.20)	0.863	0.142	48.7%	NA	
Publication year								
2019 ≥	5	69	−0.18 (−0.49, 0.12)	0.231	0.033	61.8%	NA	0.376
2019 <	1	193	−0.03 (−0.19, 0.13)	0.712	—	—	NA	
Analysis and subgroup results of Ganoderma supplementation on TC								
Overall effect	6	262	−0.18 (−0.39, 0.02)	0.083	0.190	32.8%	0.020	
Health condition								
Chronic disease	3	147	0.02 (−0.23, 0.26)	0.899	0.397	0.0%	NA	0.069
At‐risk but generally healthy individuals	1	69	−0.25 (−0.48, −0.02)	**0.033**	—	—	NA	
Healthy	2	46	−0.50 (−0.90, −0.11)	**0.013**	0.611	0.0%	NA	
Ganoderma dosage								
1400 <	2	79	−0.26 (−0.48, −0.04)	**0.020**	0.791	0.0%	NA	0.549
1400 ≤	4	183	−0.14 (−0.47, 0.19)	0.403	0.092	53.5%	NA	
Intervention duration								
8 ≥	3	115	−0.31 (−0.51, −0.12)	**0.002**	0.491	0.0%	NA	0.041
8 <	3	147	0.02 (−0.23, 0.26)	0.899	0.397	0.0%	NA	
Intervention age								
50 >	3	115	−0.31 (−0.51, −0.12)	**0.002**	0.491	0.0%	NA	0.041
50 ≤	3	147	0.02 (−0.23, 0.26)	0.899	0.397	0.0%	NA	
Geographical region								
East Asia	4	109	−0.29, (−0.55, −0.04)	**0.025**	0.545	0.0%	NA	0.071
Middle East	1	69	−0.25 (−0.48, −0.02)	**0.033**	—	—	NA	
America, Oceania and Europe	1	84	0.20 (−0.16, 0.56)	0.281	—	—	NA	
Sample size								
40 >	3	69	−0.37 (−0.69, −0.05)	**0.024**	0.461	0.0%	NA	0.199
40 ≤	3	193	−0.09 (−0.37, 0.19)	0.526	0.121	52.7%	NA	
Publication year								
2019 ≥	5	193	−0.16 (−0.44, 0.12)	0.263	0.143	41.7%	NA	0.633
2019 <	1	69	−0.25 (−0.48, −0.02)	**0.033**	—	—	NA	
Analysis and subgroup results of Ganoderma supplementation on TG								
Overall effect	6	262	−0.12 (−0.30, 0.06)	0.197	0.024	61.2%	0.025	
Health condition								
Chronic disease	3	147	0.05 (−0.51, 0.61)	0.861	0.024	73.2%	NA	0.073
At‐risk but generally healthy individuals	1	69	−0.03 (−0.16, 0.10)	0.639	—	—	NA	
Healthy	2	46	−0.24 (−0.39, −0.10)	**0.001**	0.759	0.0%	NA	
Ganoderma dosage								
1400 <	2	79	−0.12 (−0.36, 0.11)	0.304	0.103	62.5%	NA	0.773
1400 ≤	4	183	−0.06 (−0.40, 0.28)	0.716	0.029	66.7%	NA	
Intervention duration								
8 ≥	3	115	−0.16 (−0.32, 0.01)	0.060	0.083	59.9%	NA	0.489
8 <	3	147	0.05 (−0.51, 0.61)	0.861	0.024	73.2%	NA	
Intervention age								
50 >	3	115	−0.16 (−0.32, 0.01)	0.060	0.083	59.9%	NA	0.489
50 ≤	3	147	0.05 (−0.51, 0.61)	0.861	0.024	73.2%	NA	
Geographical region								
East Asia	4	109	−0.18 (−0.44, 0.09)	0.186	0.047	62.3%	NA	0.511
Middle East	1	69	−0.03 (−0.16, 0.10)	0.639	—	—	NA	
America, Oceania and Europe	1	84	0.10 (−0.40, 0.60)	0.695	—	—	NA	
Sample size								
40 >	3	69	−0.26 (−0.40, −0.13)	**< 0.001**	0.691	0.0%	NA	0.028
40 ≤	3	193	0.11 (−0.19, 0.42)	0.467	0.142	48.7%	NA	
Publication year								
2019 ≥	5	69	−0.14 (−0.38, 0.10)	0.250	0.051	57.6%	NA	0.425
2019 <	1	193	−0.03 (−0.16, 0.10)	0.639	—	—	NA	
Analysis and subgroup results of Ganoderma supplementation on ALT								
Overall effect	5	273	−2.06 (−7.64, 3.52)	0.470	< 0.001	95.1%	36.848	
Health condition								
Chronic disease	2	60	1.59 (−1.80, 4.97)	0.358	0.817	0.0%	NA	0.000
At‐risk but generally healthy individuals	1	42	−10.78 (−12.75, −8.81)	**< 0.001**	—	—	NA	
Healthy	2	171	−0.56 (−1.82, 0.71)	0.389	0.754	0.0%	NA	
Ganoderma dosage								
1400 <	3	197	−3.25 (−11.18, 4.68)	0.421	< 0.001	97.4%	NA	0.461
1400 ≤	2	76	−0.02 (−3.39, 3.36)	0.993	0.468	0.0%	NA	
Intervention duration								
8 ≥	2	56	0.34 (−3.10, 3.77)	0.848	0.346	0.0%	NA	0.380
8 <	3	217	−3.49 (−11.32, 4.34)	0.382	< 0.001	97.4%	NA	
Intervention age								
50 >	4	233	−2.81 (−9.29, 3.67)	0.395	< 0.001	96.2%	NA	0.326
50 ≤	1	40	1.20 (−3.50, 5.90)	0.617	—	—	NA	
Geographical region								
East Asia	4	253	−2.99 (−9.40, 3.41)	0.360	< 0.001	96.1%	NA	0.224
Middle East	—	—	—	—	—	—	NA	
America, Oceania and Europe	1	20	2.00 (−2.87, 6.87)	0.421	—	—	NA	
Sample size								
40 >	2	56	0.34 (3.10, 3.77)	**0.848**	0.346	0.0%	NA	0.380
40 ≤	3	217	−3.49 (−11.32, 4.34)	0.382	< 0.001	97.4%	NA	
Publication year								
2019 ≥	4	138	−2.41 (−9.86, 5.04)	0.526	< 0.001	93.2%	NA	0.620
2019 <	1	135	−0.50 (−1.81, 0.81)	0.454	—	—	NA	
Analysis and subgroup results of Chlorella supplementation on AST								
Overall effect	4	233	−1.52 (−5.09, 2.04)	0.402	< 0.001	85.6%	10.540	
Analysis and subgroup results of Ganoderma supplementation on Creatinine								
Overall effect	6	361	−0.14 (−0.27, −0.02)	**0.028**	< 0.001	80.0%	0.015	
Health condition								
Chronic disease	3	122	−0.18 (−0.53, 0.17)	0.318	0.005	81.2%	NA	0.015
At‐risk but generally healthy individuals	1	68	−0.39 (−0.57, −0.21)	**< 0.001**	—	—	NA	
Healthy	2	171	−0.04 (−0.19, 0.12)	0.644	0.030	78.7%	NA	
Ganoderma dosage								
1400 <	4	285	−0.19 (−0.34, −0.04)	**0.011**	< 0.001	85.1%	NA	0.016
1400 ≤	2	76	0.06 (−0.08, 0.20)	0.411	0.985	0.0%	NA	
Intervention duration								
8 ≥	3	118	−0.10 (−0.32, 0.13)	0.410	0.001	85.6%	NA	0.456
8 <	3	243	−0.23 (−0.50, 0.04)	0.094	0.009	78.8%	NA	
Intervention age								
50 >	3	191	−0.03 (−0.13, 0.07)	0.549	0.028	72.1%	NA	0.000
50 ≤	3	170	−0.39 (−0.52, −0.25)	**< 0.001**	0.990	0.0%	NA	
Geographical region								
East Asia	5	341	−0.19 (−0.35, −0.02)	**0.029**	< 0.001	81.9%	NA	0.075
Middle East	—	—	—	—	—	—	NA	
America, Oceania and Europe	1	20	0.00 (−0.12, 0.12)	1.000	—	—	NA	
Sample size								
40 >	2	56	0.02 (−0.07, 0.12)	0.602	0.525	0.0%	NA	0.015
40 ≤	4	305	−0.27 (−0.50, −0.05)	**0.016**	0.001	82.3%	NA	
Publication year								
2019 ≥	3	96	0.02 (−0.07, 0.12)	0.602	0.817	0.0%	NA	0.018
2019 <	3	265	−0.28 (−0.51, −0.05)	**0.019**	< 0.001	88.2%	NA	
Analysis and subgroup results of Ganoderma supplementation on FRAP								
Overall effect	4	127	−8.60 (−72.07, 54.87)	0.791	0.125	47.7%	1900	
Analysis and subgroup results of Ganoderma supplementation on GPx								
Overall effect	3	88	2.29 (1.67, 2.92)	**< 0.001**	0.986	0.0%	0.000	
Analysis and subgroup results of Ganoderma supplementation on MDA								
Overall effect	6	237	−0.07 (−0.14, 0.01)	0.071	< 0.001	96.0%	0.005	
Health condition								
Chronic disease	3	123	−0.18 (−0.38, 0.02)	0.072	0.008	79.2%	NA	0.000
At‐risk but generally healthy individuals	1	68	−0.08 (−0.10, −0.06)	**< 0.001**	—	—	NA	
Healthy	2	46	0.02 (0.02, 0.02)	**< 0.001**	0.860	0.0%	NA	
Ganoderma dosage								
1400 <	4	178	−0.10 (−0.19, −0.01)	**0.024**	< 0.001	97.6%	NA	0.064
1400 ≤	2	59	0.01 (−0.07, 0.09)	0.782	0.630	0.0%	NA	
Intervention duration								
8 ≥	3	108	−0.06 (−0.20, 0.09)	0.436	< 0.001	88.5%	NA	0.724
8 <	3	129	−0.09 (−0.19, 0.01)	0.086	0.079	60.5%	NA	
Intervention age								
50 >	2	46	0.02 (0.02, 0.02)	**< 0.001**	0.860	0.0%	NA	0.005
50 ≤	4	191	−0.14 (−0.24, −0.03)	**0.015**	0.008	74.7%	NA	
Geographical region								
East Asia	6	237	−0.07 (−0.14, 0.01)	0.071	< 0.001	96.0%	NA	
Middle East	—	—	—	—	—	—	NA	
America, Oceania and Europe	—	—	—	—	—	—	NA	
Sample size								
40 >	4	107	−0.01 (−0.09, 0.07)	0.759	0.055	60.5%	NA	0.132
40 ≤	2	130	−0.15 (−0.32, 0.01)	0.069	0.009	85.2%	NA	
Publication year								
2019 ≥	3	69	0.02 (0.02, 0.02)	**< 0.001**	0.871	0.0%	NA	0.009
2019 <	3	168	−0.19 (−0.35, −0.03)	**0.017**	0.006	80.7%	NA	
Analysis and subgroup results of Ganoderma supplementation on SOD								
Overall effect	4	163	76.86 (−21.51, 175.23)	0.126	< 0.001	94.3%	8000	
Analysis and subgroup results of Ganoderma supplementation on HR								
Overall effect	3	135	−3.92 (−7.45, −0.40)	**0.029**	0.116	53.5%	5.168	

Abbreviations: ALP, alkaline phosphatase; ALT, alanine aminotransferase; AST, aspartate aminotransferase; BMI, body mass index; CI, confidence interval; DBP, diastolic blood pressure; FRAP, ferric reducing ability of plasma; GPx, glutathione peroxidase; HDL‐C, high‐density lipoprotein cholesterol; HR, Heart rate; hs‐CRP, high‐sensitivity C‐reactive protein; IL‐6, interleukin‐6; INT, intervention group; LDL‐C, low‐density lipoprotein cholesterol; MDA, malondialdehyde; NA, not applicable; SBP, systolic blood pressure; SOD, superoxide dismutase; TC, total cholesterol; TG, triglycerides; TNF‐α, tumor necrosis factor‐alpha; WC, waist circumference; WHR, waist‐to‐hip ratio; WMD, weighted mean difference. Bold values are statistically significant.

In the health condition subgroup analysis, Ganoderma supplementation showed no significant effects in patients with chronic diseases. In at‐risk but generally healthy individuals, significant changes were observed in ALT and creatinine levels, while in healthy individuals, improvements were noted in LDL‐C and TG levels. Ganoderma supplementation significantly affected BMI, TC, and MDA in both at‐risk and healthy populations. However, no substantial effects were found on weight, DBP, SBP, fasting glucose, or HDL‐C.

In the dosage subgroup analysis, Ganoderma supplementation at doses < 1400 mg/day led to significant improvements in BMI, TC, creatinine, and MDA. In contrast, doses ≥ 1400 mg/day resulted in no significant changes.

Regarding intervention duration, Ganoderma supplementation for ≤ 8 weeks significantly improved BMI and TC, whereas interventions lasting > 8 weeks showed no significant effects.

Age subgroup analysis revealed that Ganoderma supplementation significantly improved BMI and TC in participants aged < 50 years. Among those aged ≥ 50 years, notable changes were observed in creatinine levels. Additionally, MDA levels were significantly affected in both age groups. No significant effects were observed for weight, DBP, SBP, fasting glucose, HDL‐C, LDL‐C, TG, or ALT across age subgroups.

In geographical subgroup analysis, Ganoderma supplementation improved LDL‐C and creatinine levels in East Asian participants. Studies conducted in the Middle East demonstrated significant effects on BMI, while research from America, Oceania, and Europe showed significant impacts on DBP and SBP. TC levels were influenced in both East Asian and Middle Eastern groups. No significant changes were found for weight, fasting glucose, HDL‐C, TG, ALT, or MDA in any geographical group.

For studies with < 40 participants, Ganoderma supplementation significantly improved TC, TG, and ALT levels. Conversely, studies with ≥ 40 participants showed significant effects on BMI and creatinine. No significant effects were observed for weight, DBP, SBP, fasting glucose, HDL‐C, LDL‐C, or MDA across sample size subgroups.

Subgroup analysis by publication year revealed significant improvements in TC and creatinine levels in studies published before 2019. MDA levels were significantly affected in studies conducted both before and after 2019. However, no significant changes were noted for BMI, weight, DBP, SBP, fasting glucose, HDL‐C, LDL‐C, TG, or ALT in either group.

### 3.13 GRADE Assessment

The GRADE profile for Ganoderma supplementation, summarizing the level of certainty in the outcomes, is presented in Table 4. The quality of evidence was assessed as very low across all outcomes.

**TABLE 4:** GRADE profile of Ganoderma supplementation on cardiovascular risk factors.

Outcomes	Risk of bias	Inconsistency	Indirectness	Imprecision	publication bias	Number (INT/CON)	WMD (95% CI)	Quality of evidence
BMI	Very serious^a^	Not serious	Not serious	Serious^b^	None	216/176	−0.43 (−0.77, −0.10)	⊕◯◯◯ Very low
Body fat percentage	Very serious^a^	Not serious	Serious^c^	Very serious^b^	None	93/85	−0.42 (−1.55, 0.71)	⊕◯◯◯ Very low
WC	Very serious^a^	Not serious	Serious^c^	Very serious^b^, ^d^	None	113/79	−0.45 (−1.02, 0.12)	⊕◯◯◯ Very low
Weight	Serious^a^	Not serious	Not serious	Very serious^b^, ^d^	None	179/168	−0.15 (−1.85, 1.55)	⊕◯◯◯ Very low
WHR	Very serious^a^	Not serious	Serious^c^	Very serious^b^, ^d^	None	72/59	0.00 (−0.00, 0.01)	⊕◯◯◯ Very low
DBP	Very serious^a^	Serious^e^	Serious^c^	Very serious^b^, ^d^	None	148/139	−0.72 (−3.43, 1.99)	⊕◯◯◯ Very low
SBP	Very serious^a^	Serious^e^	Serious^c^	Very serious^b^, ^d^	None	148/139	−1.50 (−4.25, 1.26)	⊕◯◯◯ Very low
Fasting glucose	Serious^a^	Not serious	Serious^c^	Very serious^b^, ^d^	None	135/101	−0.03 (−0.13, 0.07)	⊕◯◯◯ Very low
hs‐CRP	Very serious^a^	Very serious^e^	Very serious^c^	Very serious^b^, ^d^	None	69/67	−0.28 (−0.91, 0.35)	⊕◯◯◯ Very low
TNF‐α	Very serious^a^	Very serious^e^	Very serious^c^	Very serious^b^, ^d^	None	85/83	−7.70 (−27.84, 12.43)	⊕◯◯◯ Very low
HDL‐C	Very serious^a^	Not serious	Serious^c^	Very serious^b^, ^d^	None	146/116	0.02 (−0.04, 0.08)	⊕◯◯◯ Very low
LDL‐C	Serious^a^	Serious^e^	Serious^c^	Very serious^b^, ^d^	None	146/116	−0.13 (−0.34, 0.08)	⊕◯◯◯ Very low
TC	Serious^a^	Serious^e^	Serious^c^	Very serious^b^, ^d^	None	146/116	−0.18 (−0.39, 0.02)	⊕◯◯◯ Very low
TG	Very serious^a^	Serious^e^	Serious^c^	Very serious^b^, ^d^	None	146/116	−0.12 (−0.30, 0.06)	⊕◯◯◯ Very low
ALT	Serious^a^	Very serious^e^	Serious^c^	Very serious^b^, ^d^	None	141/132	−2.06 (−7.64, 3.52)	⊕◯◯◯ Very low
AST	Very serious^a^	Very serious^e^	Serious^c^	Very serious^b^, ^d^	None	121/112	−1.52 (−5.09, 2.04)	⊕◯◯◯ Very low
Creatinine	Serious^a^	Very serious^e^	Serious^c^	Serious^b^	None	185/176	−0.14 (−0.27, −0.02)	⊕◯◯◯ Very low
FRAP	Very serious^a^	Not serious	Serious^c^	Very serious^b^, ^d^	None	64/63	−8.60 (−72.07, 54.87)	⊕◯◯◯ Very low
GPx	Very serious^a^	Not serious	Serious^c^	Serious^b^	None	44/44	2.29 (1.67, 2.92)	⊕◯◯◯ Very low
MDA	Very serious^a^	Very serious^e^	Serious^c^	Very serious^b^, ^d^	None	121/116	−0.07 (−0.14, 0.01)	⊕◯◯◯ Very low
SOD	Very serious^a^	Very serious^e^	Serious^c^	Very serious^b^, ^d^	None	83/80	76.86 (−21.51, 175.23)	⊕◯◯◯ Very low
HR	Very serious^a^	Serious^e^	Serious^c^	Serious^b^	None	69/66	−3.92 (−7.45, −0.40)	⊕◯◯◯ Very low

Abbreviations: ALP, alkaline phosphatase; ALT, alanine aminotransferase; AST, aspartate aminotransferase; BMI, body mass index; CI, confidence interval; DBP, diastolic blood pressure; FRAP, ferric reducing ability of plasma; GPx, glutathione peroxidase; HDL, high‐density lipoprotein; HR, heart rate; hs‐CRP, high‐sensitivity C‐reactive protein; IL‐6, interleukin‐6; INT, intervention group; LDL, low‐density lipoprotein; MDA, malondialdehyde; SBP, systolic blood pressure; SOD, superoxide dismutase; TC, total cholesterol; TG, triglycerides; TNF‐α, tumor necrosis factor‐alpha; WC, waist circumference; WHR, waist‐to‐hip ratio; WMD, weighted mean difference.

^a^ Downgraded since more than 50% of the participants were from high‐risk bias studies.

^b^ Downgraded since the participants included were less than 400 people.

^c^ Downgraded for indirectness in the country.

^d^ Downgraded since the 95% CI crosses the threshold of interest.

^e^ The *I* ^2^ value was > 50% (or Heterogeneity among the studies was high).

## 4 Discussion

### 4.1 Summary of Findings

In this present study, the data from 17 RCTs involving 971 participants were pooled and analyzed. The findings indicated that *G. lucidum* supplementation significantly affects BMI (WMD = −0.43 kg/m^2^, 95% CI: −0.77 to −0.10, *p* = 0.011), creatinine (WMD = −0.14 mg/dL, 95% CI: −0.27 to −0.02, *p* = 0.028), GPx (WMD = 2.29 U/g Hb, 95% CI: 1.67 to 2.92, *p* < 0.001), and heart rate (WMD = −3.92 bpm, 95% CI: −7.45 to −0.40, *p* = 0.029). However, *G. lucidum* supplementation did not show significant effects on BF, WC, WHR, DBP, SBP, fasting glucose, hs‐CRP, TNF‐α, HDL‐C, LDL‐C, TC, TG, ALT, AST, FRAP, MDA, or SOD. This meta‐analysis of 17 RCTs found modest effects of *Ganoderma lucidum* supplementation on BMI, creatinine, GPx, and HR, but no significant impact on BP, lipid profile, or inflammatory markers.

To our knowledge, this systematic review and dose–response meta‐analysis is the first to employ the GRADE methodology to investigate the effects of Ganoderma supplementation on cardiometabolic outcomes in adults. The lack of significant effects on key cardiovascular risk factors such as BP, lipid profile, and inflammatory markers (hs‐CRP, TNF‐α) merits further explanation. Several factors may account for these null findings. First, the bioactive compounds in *G. lucidum* , such as polysaccharides and triterpenes, may have limited potency or bioavailability in humans compared to preclinical models, potentially insufficient to produce detectable changes in BP, lipids, or inflammation within the trial durations and dosages studied (ranging from 225 to 11,200 mg/day). For instance, triterpenes, hypothesized to inhibit ACE and reduce oxidative stress, may require higher concentrations or longer exposure to influence BP significantly, as suggested by animal studies (Shevelev et al. 2018). Similarly, polysaccharides' anti‐inflammatory effects (e.g., via cytokine modulation) observed in vitro may not translate effectively to human systemic inflammation markers like hs‐CRP or TNF‐α due to differences in metabolism or gut microbiota interactions (Chang et al. 2015). Second, the marked heterogeneity in intervention protocols—spanning dosages, formulations (e.g., extracts, spores), and durations (1–24 weeks)—likely diluted potential effects, as reflected in the moderate to high *I* ^2^ values for BP (*I* ^2^ = 74.0% for DBP, 52.4% for SBP) and inflammation (*I* ^2^ = 91.9% for hs‐CRP, 89.9% for TNF‐α). Third, the small sample sizes (most studies < 100 participants) and methodological weaknesses of the included RCTs, rated predominantly as ‘Poor’ quality with very low‐GRADE evidence, may have lacked the statistical power to detect subtle but meaningful changes in these outcomes. Finally, the baseline characteristics of participants, often including healthy or at‐risk individuals with normal BP, lipid, or inflammatory profiles, may have constrained the scope for improvement, a ceiling effect not observed in preclinical models with induced pathologies (e.g., HTN, dyslipidemia). The observed BMI reduction (−0.43 kg/m^2^) is statistically significant but falls below the clinically meaningful threshold of ≥ 0.5–1 kg/m^2^ for reducing cardiometabolic risk (Donnelly et al. 2009). Similarly, the HR reduction (−3.92 bpm) may have limited clinical impact in normotensive populations but could be relevant in hypertensive contexts. The lack of significant effects on BP, lipid profile, and fasting glucose may reflect limited bioavailability of *Ganoderma*'s bioactive compounds, insufficient trial durations, or ceiling effects in populations with normal baseline values.

The pooled results from studies examining the effects of *Ganoderma* on heart rate revealed a statistically significant decrease, with benefits evident across various dosage forms, intervention durations, and participant characteristics. The current study reported that at the conclusion of the intervention period, individuals consuming *Ganoderma* exhibited no statistically or clinically significant improvements in chronic disease markers, LDL‐C, or TG levels among the healthy population. However, supplementation significantly impacted BMI, TC, and MDA in at‐risk individuals, as well as in generally healthy individuals. No significant effects were observed on weight, DBP, SBP, fasting glucose, or HDL‐C. Notably, *Ganoderma* supplementation at doses below 1400 mg/day resulted in significant improvements in BMI, TC, creatinine levels, and MDA, whereas doses exceeding 1400 mg/day did not yield such improvements. Furthermore, the duration of intervention yielded significant enhancements in BMI and TC for participants under 50 years of age, while individuals over 50 exhibited notable changes in creatinine levels. *Ganoderma* supplementation positively influenced MDA in both age groups. The study found that *Ganoderma* supplementation for 8 weeks or less significantly improved BMI and TC, whereas interventions extending beyond 8 weeks did not produce significant changes. Gender differences were also observed, with significant enhancements in BMI and TC among participants under 50 years old, while those aged 50 and above displayed notable alterations in creatinine levels. Moreover, MDA was affected by *Ganoderma* supplementation in both groups. No significant differences were detected in weight, DBP, SBP, fasting glucose, HDL‐C, LDL‐C, TG, or ALT. The predominantly poor methodological quality of included studies, with 16 of 17 rated ‘Poor’ by the Cochrane Risk of Bias 2.0 tool, introduces significant uncertainty. The high risk of bias in randomization, blinding, and allocation concealment likely contributes to variability and limits reliability. High heterogeneity (e.g., *I* ^2^ = 80% for creatinine, 96% for MDA) likely arises from differences in study populations, intervention protocols, and geographical regions, as evidenced by subgroup analyses showing regional variations in DBP effects. Our findings corroborate previous research indicating that *Ganoderma* supplementation did not exhibit significant effects on BF, WC, weight, WHR, DBP, SBP, fasting glucose, hs‐CRP, TNF‐α, HDL‐C, LDL‐C, TC, TG, ALT, AST, FRAP, and MDA (Hu et al. 2014; Babamiri et al. 2022b; Klupp et al. 2016; Chu et al. 2012).

Our meta‐analysis identified statistically significant effects of *G. lucidum* supplementation on several outcomes, including BMI (WMD = −0.43 kg/m^2^, 95% CI: −0.77 to −0.10, *p* = 0.011), creatinine (WMD = −0.14 mg/dL, 95% CI: −0.27 to −0.02, *p* = 0.028), GPx (WMD = 2.29 U/g Hb, 95% CI: 1.67–2.92, *p* < 0.001), and heart rate (WMD = −3.92 bpm, 95% CI: −7.45 to −0.40, *p* = 0.029). However, the clinical meaningfulness of these effect sizes warrants careful consideration. For instance, the reduction in BMI (−0.43 kg/m^2^) is statistically significant but modest, falling below the threshold of ≥ 0.5–1 kg/m^2^ often cited as clinically relevant for improving cardiometabolic risk in overweight or obese individuals (Donnelly et al. 2009). Similarly, the decrease in heart rate (−3.92 bpm) may not substantially alter cardiovascular risk in normotensive populations, though it could hold potential relevance in hypertensive patients, pending further study. The reduction in creatinine (−0.14 mg/dL) is small relative to normal reference ranges (0.6–1.2 mg/dL), suggesting minimal impact on renal function, while the increase in GPx (2.29 U/g Hb), an antioxidant enzyme, is promising but lacks established benchmarks for clinical significance in oxidative stress reduction. Notably, no significant effects were observed for critical cardiovascular and metabolic markers such as BP, lipid profile, or fasting glucose, and the very low quality of evidence across all outcomes (GRADE assessment, Table 4) underscores the uncertainty surrounding these findings. Given the modest effect sizes and very low quality of evidence, recommending *G. lucidum* supplementation is premature. However, its safety profile and potential benefits in specific populations (e.g., overweight individuals) suggest a role as a complementary therapy pending further evidence.

### 4.2 RCTs of *G. lucidum*

Over the past few years, clinical trials involving Ganoderma have been conducted to evaluate its efficacy in treating various disorders. In 1990, a water‐soluble extract of *G. lucidum* was employed to assess its inhibitory effects on platelet aggregation in patients with atherosclerotic conditions. The maximal platelet aggregation inhibition rate was significantly reduced compared to the placebo group following 2 weeks of oral treatment with *G. lucidum* extract (Jun and Ke‐yan 1990). However, this extract was not formulated as a novel pharmacological agent for atherosclerotic disease. The studies have elucidated the potential therapeutic applications of *G. lucidum* . The polysaccharides derived from Ganoderma have undergone seven clinical trials addressing chronic hepatitis B, advanced lung cancer, T2DM, CHD, and neurasthenia (Gao, Chen, et al. 2004; Gao et al. 2003; Gao, Zhou, Chen, Dai, and Ye 2002; Gao, Zhou, Chen, Dai, Ye, and Gao 2002; Zhou et al. 2005). Clinical findings indicated that the polysaccharides of *G. lucidum* were well tolerated and were orally administered to patients with advanced cancer over a period of 12 weeks, with no significant side effects reported (Gao, Zhou, Chen, Dai, Ye, and Gao 2002). Although the clinical trials involving *G. lucidum* did not yield successful outcomes, the findings provide valuable insights for the future development of Ganoderma‐related medications, particularly regarding the necessity of identifying suitable biomarkers, endpoints, and pharmacological processes (Zhou et al. 2005).

A double‐blind (DB), randomized (R), placebo‐controlled (PC) trial assessing the efficacy of Ganoderma water extract in patients with RA was unsuccessful; the extract demonstrated no significant effects on anti‐inflammatory responses and immune regulation, although it was deemed safe and well tolerated (Li et al. 2007). Additionally, a further DB experiment conducted in Australia aimed at treating cardiovascular disorders in individuals with MetS or diabetes was unsuccessful (Klupp et al. 2016). In summary, it is suggested that Ganoderma extracts or polysaccharides do not exhibit substantial bioactivities in contemporary clinical studies. Recent investigations indicate that the bioactivities of water extracts from Ganoderma are associated with the composition of gut flora; thus, the formulation of Ganoderma‐based pharmaceuticals remains complex due to intricate biological pathways (Chang et al. 2015). To date, no Ganoderma‐related pharmaceuticals have received approval from the U.S. Food and Drug Administration (FDA), likely due to the ambiguity surrounding its active pharmaceutical ingredients and mechanisms of action.

Antioxidants are potential therapeutic agents that may contribute to the prevention of atherosclerosis and various other disorders. Numerous in vitro and in vivo studies have demonstrated that components of *G. lucidum* possess antioxidant properties; however, evidence supporting these effects in human subjects has been lacking. The subsequent research investigated the effects of *G. lucidum* supplementation on several parameters, including antioxidant biomarker status and the risk of CHD. A DB, crossover, PC intervention study was conducted involving healthy individuals. Following a 10‐day treatment period, there was an observed increase in plasma total antioxidant indicators and an improvement in biomarkers associated with CHD. Importantly, no evidence of renal, hepatic, or deoxyribonucleic acid (DNA) toxicity was noted (Wachtel‐Galor, Szeto, et al. 2004). A crossover human intervention study involving seven healthy individuals was conducted to assess the antioxidant capacity of *G. lucidum* , revealing an increase in plasma total antioxidant power following a single dosage of *G. lucidum* extract (Wachtel‐Galor et al. 2010). Additionally, the antioxidant capabilities of *G. lucidum* polysaccharide peptide (PsP) were evaluated. A clinical trial was performed involving patients at high risk and those with stable angina, who received PsP for 90 days. The polysaccharides exhibited significant antioxidant properties in the context of atherosclerosis among both high‐risk and stable cohorts. Patients treated with PsP demonstrated elevated SOD levels, decreased MDA levels, and a reduction in the numbers of circulating endothelial cells and endothelial progenitor cells (Sargowo et al. 2019). A DB, R, multicenter trial was conducted to assess the effects and safety of *G. lucidum* polysaccharides in patients with CHD. Eighty‐eight patients in the experimental group received *G. lucidum* polysaccharides for a duration of 12 weeks. The administration of polysaccharides resulted in significant improvements in patient outcomes, including reductions in BP and serum cholesterol levels (Gao, Chen, et al. 2004).

Further research indicated PsP derived from *G. lucidum* exhibited anti‐inflammatory properties and conferred protection to vascular endothelial cells in patients with ST‐elevation myocardial infarction and non‐ST‐elevation myocardial infarction who presented with dyslipidemia risk factors (Sargowo et al. 2019). An RCT was conducted to assess the efficacy of *G. lucidum* in addressing cardiovascular risk factors associated with metabolic syndrome. The results indicated that the administration of *G. lucidum* over a 16‐week period did not significantly affect hemoglobin A1c(HbA1c) or fasting plasma glucose (FPG) levels. Furthermore, the utilization of *G. lucidum* was associated with an increased risk of certain moderate adverse events, including headache, fatigue, and gastrointestinal issues (Klupp et al. 2016). However, none of the aforementioned human studies reported significant adverse effects, thereby highlighting *G. lucidum* as a potential therapeutic and preventive agent for various CVDs. A study involving 37 individuals at heightened risk for atherosclerosis, who were undergoing standard CVD treatment, demonstrated that *G. lucidum* PsP significantly reduced levels of hs‐CRP, IL‐6, TNF‐α, and MDA (Widya et al. 2016).

Additionally, a study involving 34 patients with stable angina pectoris demonstrated that the administration of PsP significantly reduces TC levels (Ubaidillah et al. 2016). The efficacy of *G. lucidum* in CVDs was assessed in a prospective, DB, PC study. Eighty‐four volunteers diagnosed with T2DM and MetS received supplements of *G. lucidum* , or *G. lucidum* combined with Cordyceps sinensis, or a placebo to address cardiovascular risk factors. The results of the study indicated that *G. lucidum* did not provide any significant advantages in the prevention of CVD among individuals with MetS (Klupp et al. 2016). An analysis of five studies encompassing 398 participants indicated that *G. lucidum* was ineffective in the treatment of elevated BP (Klupp et al. 2015). Collectively, these findings suggest that *G. lucidum* is most effective in providing cardiovascular protection when used in conjunction with cardiovascular medications. Furthermore, Ganoderma pharmaceuticals have been utilized either as monotherapy or in combination with other compounds or chemotherapeutic agents in clinical studies. However, the composition of these prospective Ganoderma pharmaceuticals remains complex and necessitates further investigation. This complexity presents a significant challenge for clinical trials, which must be conducted in accordance with FDA guidelines on Chemistry, Manufacturing, and Controls (CMC) for clinical studies. Consequently, a transparent active pharmaceutical ingredient (API) for candidate pharmaceuticals is essential for future clinical investigations of Ganoderma.

The systematic review reveals significant methodological limitations across the included RCTs that fundamentally compromise the reliability and generalizability of the findings. A critical examination of the study quality assessment table exposes pervasive methodological vulnerabilities: the overwhelming majority of studies (16 out of 17) were rated as “Poor” quality, with only one study achieving a “Fair” rating. The most prominent methodological deficiencies include inconsistent and often inadequate approaches to randomization, allocation concealment, and blinding. Specifically, many studies exhibited unclear (U) or high‐risk (H) domains in crucial methodological areas such as random sequence generation and allocation concealment. The blinding processes were particularly problematic, with numerous studies showing incomplete or unclear blinding of participants, personnel, and outcome assessors—a critical weakness that introduces significant potential for performance and detection bias.

The geographical concentration of studies presents another substantial methodological constraint, with 12 out of 17 studies conducted in East Asian countries, predominantly in China, Hong Kong, and Taiwan. This geographical homogeneity severely limits the external validity and generalizability of the findings to diverse populations with different genetic, dietary, and environmental contexts. Furthermore, the studies demonstrated marked heterogeneity in intervention protocols, including substantial variations in *G. lucidum* dosage (ranging from 225 to 11,200 mg/day), supplement formulations, and trial durations (1–24 weeks). Sample sizes were consistently small, with most studies involving fewer than 100 participants, further diminishing the statistical power and reliability of the results. The predominance of short‐duration trials restricts meaningful conclusions about long‐term effects, while the lack of standardization in *G. lucidum* preparation—including variations in extraction methods, active compound concentrations, and supplement types—introduces additional layers of methodological complexity and potential bias. These systemic methodological weaknesses underscore the urgent need for more rigorous, standardized, and methodologically robust research to definitively establish the clinical significance of *G. lucidum* supplementation.

### 4.3 Animal Model for *G. lucidum* Evaluation

The present review indicates that the majority of preclinical studies involving *Ganoderma* have focused on animal models rather than human subjects. The cardioprotective properties of *G. lucidum* extract were investigated through the application of global ischemia and reperfusion in isolated and perfused rat hearts. In this study, *G. lucidum* extract was administered prophylactically to rats over a period of 15 days. This intervention was found to prevent necrotic death of rat cardiomyocytes and reduce reperfusion contracture (Lasukova et al. 2015). Importantly, the administration of *G. lucidum* extract was observed to alleviate diastolic dysfunction and prevent irreversible damage to cardiomyocytes (Lasukova et al. 2008). Diabetes mellitus, a metabolic disorder, is associated with an increased incidence of CVDs, primarily due to arterial damage resulting from elevated blood glucose levels (Klein et al. 2002; Fox et al. 2008; Shah et al. 2008).

Consequently, a supplement that supports diabetes management could significantly reduce the risk of CVD. In a study, 35 rats were administered a supplement of polysaccharides derived from *G. lucidum* extracts, which facilitated the endothelial healing process. The results indicated that polysaccharide therapy ameliorated vascular damage in this rat model (Heriansyah et al. 2019). A diet rich in lipids is recognized as a significant risk factor for the onset of CVDs (Fleming 2002), and weight reduction is a crucial strategy for preventing CVD in individuals with obesity (Sowers 2003). The aqueous extract of *G. lucidum* has been shown to diminish body weight, inflammation, and insulin resistance in mice subjected to a high‐fat diet (HFD) (Chang et al. 2015). In a separate trial, Ganoderma spores were administered orally for 4 weeks to adult male rats. These spores reduced TC and TG in diabetic rats and mitigated oxidative stress levels; notably, there was an upregulation of genes associated with lipid metabolism, specifically acyl‐CoA oxidase 1 (ACOX1), acetyl‐CoA carboxylase (ACC), and Insig‐1/2 gene expression. ACOX1 was activated over fivefold in the spore‐treated diabetic rats, indicating enhanced beta‐oxidation of lipids in these subjects (Wang et al. 2014). Dyslipidemia is a significant risk factor for CVD (Miller 2009).

In experimental animal investigations, *G. lucidum* has exhibited anti‐hyperlipidemic effects by decreasing plasma levels of TC, LDL‐C, and TG (Chen et al. 2005). Hydrogen peroxide free radicals are frequently elevated in the context of dyslipidemia, thereby increasing the risk of atherosclerosis; consequently, antioxidants are crucial for preventing vascular damage. Research involving rats on a high‐cholesterol diet supplemented with 50, 150, and 300 mg/kg body weight of PsP derived from *G. lucidum* demonstrated that PsP acted as a potent antioxidant. Furthermore, PsP may inhibit the atherogenesis associated with dyslipidemia, with the optimal dosage identified as 300 mg/kg body weight (Wihastuti and Heriansyah 2017). Additional studies indicated that polysaccharides extracted from *G. lucidum* significantly reduced body weight gain in mice subjected to an HFD, underscoring its potential role as a hypolipidemic agent. Moreover, it exhibited antioxidant and antiapoptotic properties in HFD‐fed mice (Liang et al. 2018). A study involving adult male hypertensive rats revealed that the administration of water extracts from *G. lucidum* for 7 weeks resulted in BP reductions comparable to those observed in rats treated with losartan, an angiotensin II receptor antagonist (Shevelev et al. 2018).

### 4.4 Future Approach in RCTs of *G. lucidum*

Fifteen clinical studies involving Ganoderma have been registered; however, only five are currently in progress, with the majority either not recruiting participants or having an unspecified status. The five ongoing clinical trials focus on conditions such as eczema, uveitis, prostate cancer, lung cancer, breast cancer, gastrointestinal cancer, and Parkinson's disease. Table S3 provides detailed information regarding the recruitment status and the unknown status of the clinical studies.

The most recent updates regarding the quantity and quality of clinical trials fail to elucidate the intentional application of Ganoderma in the specified disorders. Although some clinical trials exist, the sample sizes, standards, procedures, and criteria employed are insufficient to meet the benchmarks established by various assessment scales (Berger and Alperson 2009). There is significant variability in trial designs, with the majority being preliminary (Phase I/II), primarily conducted to assess safety and dosage range selection for potential further studies. The authors advocate for a more rigorous evaluation, as demonstrated in the quality of clinical trials involving natural products in cancer research (Ahmad 2020), which may provide valuable insights for future clinical studies. Advanced studies (Phase III/IV) utilizing systematic methodologies, risk‐based monitoring, larger sample sizes, appropriate inclusion and exclusion criteria, established standards, blinding, randomization, dropout protocols, and multicenter trials are essential. Comprehensive literature data is crucial to substantiate the utilization of Ganoderma in conjunction with various foods, beverages, or herbs that may either antagonize or synergize its effects through mechanisms that influence absorption, bioavailability, excretion, or complex formation due to the presence of fibers in certain products. The most frequently consumed meals and beverages have been associated with reported interactions (Bushra et al. 2011).

Several products derived from Ganoderma are available in the market, including syrups, powders, and capsules. While these products may specify the quantity of active components in the recommended dosage, geographical location may be influenced by the aforementioned factors. Variability in sample characteristics can affect the quality and quantity of mycochemicals in Ganoderma, thereby impacting the active drug content in a specified dosage and subsequently the medicinal efficacy of the final product. It is imperative to assess and maintain consistency in the quality of Ganoderma samples in accordance with local variations. This can be effectively accomplished by adhering to WHO standards for the assessment and maintenance of the quality of herbal products (WHO G 2007). The findings may be robustly validated using preclinical models followed by rigorous clinical studies. The incorporation of nanoparticles (NPs) in conjunction with Ganoderma represents an innovative approach in herbal therapy aimed at enhancing therapeutic efficacy, reducing the frequency of administration, and minimizing adverse effects or toxicities. Herbal medications or extracts are frequently insoluble or possess limited solubility, potentially leading to decreased absorption, bioavailability, and therapeutic efficacy. NPs, including polymeric NPs, nanoemulsions, liposomes, nanogels, and solid lipid NPs, offer the advantage of transforming and utilizing drugs with suboptimal therapeutic properties by enhancing their solubility, bioavailability, and targeted delivery methods (Ansari et al. 2012). Comprehensive research is necessary for the extracts, powders, syrups, capsules, and isolated active compounds of Ganoderma to establish them as viable medication candidates with improved therapeutic and targeted attributes at minimal dosages and reduced toxicity.

As awareness of healthcare increases, Ganoderma‐related products have garnered significant attention due to substantial scientific evidence supporting their health benefits, including gut microbiota regulation, immunomodulation, and neuroprotection (Chang et al. 2015; Gokce et al. 2015). Additionally, ganoderic acids and β‐glucan have been recognized for their contributions to the health advantages of Ganoderma (Ferreira et al. 2015; Wu et al. 2013). Various methodologies for the production of these beneficial metabolites have been identified; therefore, Ganoderma nutraceuticals are well developed. Despite Ganoderma being widely acknowledged as a medicinal fungus, clinical trials involving Ganoderma have consistently failed over the past several decades. Although Ganoderma spp. demonstrate numerous pharmacological properties in scientific research, a significant disparity exists between nutraceutical and pharmaceutical applications. The complexity of formulation, unclear active components, and biological mechanisms have resulted in the failure of Ganoderma‐based pharmaceuticals in clinical trials. Consequently, the delineation of distinct active pharmaceutical ingredients and their pharmacological effects is essential for the advancement of Ganoderma‐derived medications.

A notable feature of this meta‐analysis is the high heterogeneity observed in certain outcomes, such as hs‐CRP (*I* ^2^ = 91.9%), TNF‐α (*I* ^2^ = 89.9%), and DBP (*I* ^2^ = 74.0%), which warrants consideration of its underlying sources. Our subgroup analyses revealed that factors such as health condition, geographical region, and sample size may contribute to this variability. For instance, DBP effects varied significantly by region (*p* = 0.013), with stronger reductions in America/Oceania/Europe (WMD = −5.20, *p* = 0.001) compared to East Asia (WMD = 0.67, *p* = 0.884, *I* ^2^ = 80.0%), potentially reflecting differences in dietary patterns, genetic profiles, or study design rigor. Similarly, the pronounced heterogeneity in inflammatory markers likely stems from clinical diversity—as well as baseline inflammation levels—and methodological factors, including assay sensitivity and timing of measurements, as seen in prior nutraceutical research (Jafari et al. 2024; Jafari et al. 2025). Sensitivity analyses further highlighted the influence of individual studies, such as Klupp et al. (2016) on total cholesterol (*I* ^2^ reduced post‐exclusion), underscoring the role of specific trial characteristics. By employing a random‐effects model and comprehensive subgroup stratification, we accounted for this variability, ensuring robust pooled estimates. These findings not only contextualize the therapeutic potential of *Ganoderma lucidum* but also highlight the complexity of its effects across diverse populations, offering a springboard for future research into personalized nutritional interventions.

### 4.5 Strengths and Limitations

This systematic review and meta‐analysis represent a pioneering effort to assess the clinical effects of *G. lucidum* supplementation on diverse health‐related indices. By employing the PRISMA guidelines and the GRADE framework, this study ensures methodological transparency and rigor, providing a robust synthesis of current evidence. The inclusion of a wide range of health outcomes, such as oxidative stress markers, inflammatory biomarkers, and lipid profiles, offers comprehensive insights into the potential therapeutic applications of *G. lucidum* . Subgroup analyses and dose–response evaluations further enrich the findings, revealing nuanced relationships between supplementation dose, duration, and health outcomes.

Notably, this analysis incorporates studies across multiple health conditions, including asthma, rheumatoid arthritis, and metabolic disorders, broadening its clinical applicability. Additionally, the exploration of plausible biological mechanisms—such as antioxidant and anti‐inflammatory pathways—enhances the translational value of the findings.

However, the study is not without limitations. The small sample sizes in several included trials and the heterogeneity in study designs, supplementation forms, and outcome measures reduce the overall strength of the evidence. A significant proportion of the trials were conducted in East Asia, which may limit the generalizability of findings to populations with different dietary, genetic, and environmental contexts. Moreover, the predominance of short‐duration studies restricts conclusions about long‐term effects. The GRADE assessment revealed a predominance of low or very low‐quality evidence for many outcomes, highlighting the need for better‐designed trials to strengthen confidence in the findings. Additionally, variability in the quality and standardization of *G. lucidum* products across studies introduces potential biases.

The GRADE assessment revealed critical methodological challenges that fundamentally undermine the certainty of evidence across all outcomes. The predominant “very low” quality of evidence stems from multiple interconnected factors: a substantial proportion of studies (> 50%) exhibited high risk of bias, significantly limiting the reliability of reported results. Methodological inconsistencies were evident through high heterogeneity (*I* ^2^ > 50%) in several outcomes, indicating substantial variability in study designs, intervention protocols, and population characteristics. Imprecision further compromised the evidence quality, with most analyses including fewer than 400 participants and confidence intervals frequently crossing the threshold of statistical significance. Indirectness was introduced by the geographical concentration of studies, predominantly in East Asian contexts, which potentially restricts the generalizability of findings. The narrow confidence intervals and the systematic downgrading across domains reflect the current limitations in research methodology surrounding *G. lucidum* supplementation. These methodological constraints necessitate a cautious interpretation of the results and underscore the urgent need for large‐scale, methodologically robust randomized controlled trials that can provide more definitive evidence regarding the clinical effects of *G. lucidum* .

An additional limitation is the absence of meta‐regression to further explore sources of heterogeneity observed across outcomes. As per the Cochrane Handbook for Systematic Reviews of Interventions (Higgins 2008), meta‐regression is not recommended when fewer than 10 studies are available per covariate due to insufficient statistical power and the risk of overfitting, which could yield unreliable results. In our analysis, the number of studies per outcome ranged from 3 to 9, falling below this threshold for all variables. Consequently, we relied on subgroup analyses stratified by factors such as health condition, dosage, and duration to investigate heterogeneity, a more feasible approach given the limited number of studies. While this choice aligns with established guidelines, it restricts our ability to simultaneously assess multiple sources of variability, a limitation that could be addressed in future meta‐analyses with larger datasets.

To address the limitations identified in this review, future research should prioritize large‐scale, multicenter RCTs involving diverse populations across different geographic and cultural contexts. These trials should employ standardized methodologies for intervention protocols, outcome measurements, and reporting standards to ensure comparability and reproducibility. The evaluation of *G. lucidum* 's long‐term safety and efficacy through extended follow‐up periods is particularly critical for understanding its role in chronic disease management.

Further research should focus on elucidating the mechanisms underlying *G. lucidum* 's health benefits by incorporating biomarker analyses and advanced molecular techniques. Trials investigating synergistic or antagonistic interactions between *G. lucidum* and other dietary components, herbs, or medications are essential for optimizing its use in clinical practice. The inclusion of advanced analytical methods, such as NP‐based formulations, could enhance the bioavailability and therapeutic potential of *G. lucidum* , paving the way for innovative applications in integrative medicine.

Finally, standardization of *G. lucidum* products in terms of active compound content, quality, and consistency should be prioritized. Regulatory frameworks, such as WHO guidelines for herbal medicines, can provide a basis for ensuring the reliability and safety of these products in clinical settings. By addressing these research gaps, future studies can significantly advance our understanding of *G. lucidum* 's therapeutic potential and inform evidence‐based recommendations for its use in health promotion and disease management. Limitations include high heterogeneity in outcomes (e.g., *I* ^2^ = 96% for MDA), small sample sizes (most studies < 100 participants), and variability in *Ganoderma* preparations (e.g., extracts vs. spore powder), which introduce potential biases and limit comparability. The geographical concentration of studies in East Asia restricts generalizability. The absence of meta‐regression, due to insufficient studies per outcome, limits exploration of heterogeneity sources.

## 5 Conclusion

This systematic review and meta‐analysis highlight the potential benefits of *G. lucidum* supplementation in improving key health‐related indices, particularly BMI, creatinine levels, GPx activity, and HR. Despite its minimal impact on other cardiometabolic parameters, the findings underscore its potential as a complementary therapy for managing select risk factors associated with cardiovascular and metabolic disease. With its favorable safety and accessibility, *G. lucidum* could be considered in dietary and therapeutic strategies. Future research should prioritize well‐designed clinical trials to explore long‐term efficacy, optimal dosage, and formulations, as well as underlying mechanisms. *Ganoderma lucidum* may have modest effects on BMI, creatinine, GPx, and heart rate, but the very low quality of evidence precludes strong recommendations. Well‐designed RCTs are needed to confirm its efficacy and clinical relevance.

## Author Contributions

**Ali Jafari:** conceptualization (equal), data curation (equal), formal analysis (equal), investigation (equal), methodology (equal), project administration (equal), resources (equal), software (equal), validation (equal), visualization (equal), writing – original draft (equal), writing – review and editing (equal). **Helia Mardani:** conceptualization (equal), data curation (equal), resources (equal), visualization (equal), writing – original draft (equal), writing – review and editing (equal). **Zahra Mirzaei Fashtali:** data curation (equal), project administration (equal), validation (equal), writing – original draft (equal), writing – review and editing (equal). **Bahareh Arghavan:** conceptualization (equal), data curation (equal), formal analysis (equal), funding acquisition (equal), investigation (equal), methodology (equal), project administration (equal), resources (equal), software (equal), supervision (equal), validation (equal), visualization (equal), writing – original draft (equal), writing – review and editing (equal).

## Ethics Statement

This study received approval from the Abadan University of Medical Sciences Ethical Committee (IR.ABADANUMS.REC.1403.128, available at (https://ethics.research.ac.ir/EthicsProposalView.php?&code=IR.ABADANUMS.REC.1403.128)). The authors have completely observed ethical issues (including plagiarism, informed consent, misconduct, data fabrication and/or falsification, double publication and/or submission, redundancy, etc.).

## Conflicts of Interest

The authors declare no conflicts of interest.

## Supporting information


Data S1

## Data Availability

All relevant data are provided within the manuscript and Supporting Information. Additionally, data analyzed for this study are available upon request from the corresponding author.
