# Supplementary material for: The Nutritional Significance of Ganoderma lucidum on Human Health: A GRADE‐Assessed Systematic Review and Meta‐Analysis of Clinical Trials
Source: Food Sci Nutr. 2025 Jun 12;13(6):e70423. doi: 10.1002/fsn3.70423 (PMC12160064; doi:10.1002/fsn3.70423)
Supplement: Supplementary file 1 — Data S1 [file FSN3-13-e70423-s001.docx]

**The Beneficial Effects of *Ganoderma lucidum* (Reishi) on Health-Related Indices: A GRADE-Assessed Systematic Review and Meta-Analysis**

*Supplementary* materials**:** *Supplementary* Tables 1-3, *Supplementary* Figure 1-9, and Supplementary References.

| **Supplementary Table 1.** Search strategy to find potential eligible randomised controlled trials (August 2024) | |
| --- | --- |
| **Groups** | **Descriptors** |
| Intervention | “Ganoderma” OR “G.lucidum” OR “Ganoderma lucidum” OR “Lingzhi” OR “Reishi” OR “mannentake” OR “Boletus lucidus” OR “Youngzhi” OR “Agaricomycetes” |
| Design | Intervention OR “Intervention Study” OR “Intervention Studies” OR “controlled trial” OR random* OR placebo OR “clinical trial” OR Trial OR “randomized controlled trial” OR “randomized clinical trial” OR RCT OR blinded OR “double blind” OR “double blinded” OR trial* OR “Pragmatic Clinical Trial” OR “Cross-Over Studies” OR “Cross-Over” OR “Cross-Over Study” OR parallel OR “parallel study” OR “parallel trial” |

**PubMed**

Number of localized studies: 324

|  | **Descriptors** | **Number of studies reached** |
| --- | --- | --- |
| #1 | "Ganoderma"[Mesh] OR "Ganoderma"[Title/Abstract] OR "G.lucidum"[Title/Abstract] OR "Ganoderma lucidum"[Title/Abstract] OR "Lingzhi"[Title/Abstract] OR "Reishi"[Title/Abstract] OR "mannentake"[Title/Abstract] OR "Boletus lucidus"[Title/Abstract] OR "Youngzhi"[Title/Abstract] OR "Agaricomycetes"[Title/Abstract] | 4,546 |
| #2 | Intervention[Title/Abstract] OR "Intervention Study"[Title/Abstract] OR "Intervention Studies"[Title/Abstract] OR "controlled trial"[Title/Abstract] OR random*[Title/Abstract] OR placebo[Title/Abstract] OR "clinical trial"[Title/Abstract] OR Trial[Title/Abstract] OR "randomized controlled trial"[Title/Abstract] OR "randomized clinical trial"[Title/Abstract] OR RCT[Title/Abstract] OR blinded[Title/Abstract] OR "double blind"[Title/Abstract] OR "double blinded"[Title/Abstract] OR trial*[Title/Abstract] OR "Pragmatic Clinical Trial"[Title/Abstract] OR "Cross-Over Studies"[Title/Abstract] OR "Cross-Over"[Title/Abstract] OR "Cross-Over Study"[Title/Abstract] OR parallel[Title/Abstract] OR "parallel study"[Title/Abstract] OR "parallel trial"[Title/Abstract] OR "Clinical Trial" [Publication Type] OR "Randomized Controlled Trial" [Publication Type] OR "Controlled Clinical Trial" [Publication Type] OR "Random Allocation"[Mesh] OR "Randomized Controlled Trials as Topic"[Mesh] OR "Pragmatic Clinical Trial" [Publication Type] OR "Pragmatic Clinical Trials as Topic"[Mesh] OR "Double-Blind Method"[Mesh] OR "Single-Blind Method"[Mesh] OR "Cross-Over Studies"[Mesh] | 3,700,936 |
| #3 | #1 AND #2 | 324 |

**Web of Science**

Number of localized studies: 567

|  | **Descriptors** | **Number of studies reached** |
| --- | --- | --- |
| #1 | TS=(“Ganoderma” OR “G.lucidum” OR “Ganoderma lucidum” OR “Lingzhi” OR “Reishi” OR “mannentake” OR “Boletus lucidus” OR “Youngzhi” OR “Agaricomycetes”) | 8,766 |
| #2 | TS=(Intervention OR “Intervention Study” OR “Intervention Studies” OR “controlled trial” OR random* OR placebo OR “clinical trial” OR Trial OR “randomized controlled trial” OR “randomized clinical trial” OR RCT OR blinded OR “double blind” OR “double blinded” OR trial* OR “Pragmatic Clinical Trial” OR “Cross-Over Studies” OR “Cross-Over” OR “Cross-Over Study” OR parallel OR “parallel study” OR “parallel trial”) | 6,440,497 |
| #3 | #1 AND #2 | 567 |

**Scopus**

Number of localized studies: 877

|  | **Descriptors** | **Number of studies reached** |
| --- | --- | --- |
| #1 | TITLE-ABS-KEY (“Ganoderma” OR “G.lucidum” OR “Ganoderma lucidum” OR “Lingzhi” OR “Reishi” OR “mannentake” OR “Boletus lucidus” OR “Youngzhi” OR “Agaricomycetes”) | 9,775 |
| #2 | TITLE-ABS-KEY (intervention OR "Intervention Study" OR "Intervention Studies" OR "controlled trial" OR random* OR placebo OR "clinical trial" OR trial OR "randomized controlled trial" OR "randomized clinical trial" OR rct OR blinded OR "double blind" OR "double blinded" OR trial* OR "Pragmatic Clinical Trial" OR "Cross-Over Studies" OR "Cross-Over" OR "Cross-Over Study" OR parallel OR "parallel study" OR "parallel trial") | 7,990,832 |
| #3 | #1 AND #2 | 877 |

**Embase**

Number of localized studies: 2,692

|  | **Descriptors** | **Number of studies reached** |
| --- | --- | --- |
| #1 | 'ganoderma'/exp OR 'ganoderma':ab,ti OR 'g.lucidum':ab,ti OR 'ganoderma lucidum':ab,ti OR 'lingzhi':ab,ti OR 'reishi':ab,ti OR 'mannentake':ab,ti OR 'boletus lucidus':ab,ti OR 'youngzhi':ab,ti OR 'agaricomycetes':ab,ti | 6,000 |
| #2 | 'randomized controlled trial'/exp OR 'randomized controlled trial (topic)'/exp OR 'pragmatic trial'/exp OR 'clinical trial'/exp OR 'clinical trial (topic)'/exp OR 'intervention study'/exp OR 'controlled study'/exp OR 'controlled clinical trial'/exp OR 'double blind procedure'/exp OR 'single blind procedure'/exp OR 'crossover procedure'/exp OR 'parallel design'/exp | 11,832,686 |
| #3 | #1 AND #2 | 2,692 |

**Cochrane**

Number of localized studies: 148

Limits: TRIALS

|  | **Descriptors** | **Number of studies reached** |
| --- | --- | --- |
| #1 | (“Ganoderma” OR “G.lucidum” OR “Ganoderma lucidum” OR “Lingzhi” OR “Reishi” OR “mannentake” OR “Boletus lucidus” OR “Youngzhi” OR “Agaricomycetes”):ti,ab,kw | 148 |

| **Supplementary Table 2.** A summary of excluded articles after full text review | |
| --- | --- |
| **Author, Year (Ref.)** | **Reason** |
| Soo, 1996 (1) | Due to Not interested study design |
| Gao, 2002 (2) | Due to Not interested study design |
| Gao, 2003 (3) | Due to Not interested study design |
| Gao, 2003 (4) | Due to Not interested study design |
| Gao, 2004 (5) | Due to Not interested study design |
| Wachtel-Galor, 2004 (6) | Duplicate |
| Zhang, 2004 (7) | Animal study |
| Gao, 2005 (8) | Due to Not interested study design |
| Kwok, 2005 (9) | Due to Not interested outcome |
| Tang, 2005 (10) | Due to Not interested outcome |
| Xi Bao, 2006 (11) | Due to Not interested study design |
| Hijikata, 2007 (12) | Due to Not interested study design |
| Wicks, 2007 (13) | Due to Not interested outcome |
| Noguchi, 2008 (14) | Due to Not interested outcome |
| Noguchi, 2008 (15) | Due to Not interested outcome |
| Wang, 2008 (16) | Due to not access full text |
| Zhuang, 2009 (17) | Due to Not interested outcome |
| Oka, 2010 (18) | Due to Not interested outcome |
| Rubel, 2011 (19) | Animal study |
| Chu, 2012 (20) | Duplicate |
| Zhuang, 2012 (21) | Due to Not interested outcome |
| Rossi, 2014 (22) | Due to Not interested outcome |
| Klupp, 2015 (23) | Review study |
| Liu, 2015 (24) | Due to Not interested study design |
| Sarker, 2015 (25) | Animal study |
| Klupp, 2016 (26) | Duplicate |
| Sargowo, 2016 (27) | Abstract |
| Tawasri, 2016 (28) | Due to Not interested outcome |
| Wu, 2016 (29) | Animal study |
| Chiu, 2017 (30) | Duplicate |
| Farikh, 2017 (31) | Due to Not interested study design |
| Putthapiban, 2017 (32) | Due to Not interested study design |
| Tsuk, 2017 (33) | Due to Not interested outcome |
| Ali, 2018 (34) | Due to Not interested study design |
| Jung, 2018 (35) | Animal study |
| Henao, 2018 (36) | Due to Not interested study design |
| Sargowo, 2018 (37) | Not interested study design |
| Adrian, 2019 (38) | Abstract |
| Nugroho, 2019 (39) | Abstract |
| Parravano, 2019 (40) | Due to Not interested outcome |
| Peltzer, 2019 (41) | Due to Not interested study design |
| Pratama, 2019 (42) | Abstract |
| Sugita, 2019 (43) | Abstract |
| Grammatikopoulou, 2020 (44) | Review study |
| Liu, 2020 (45) | Due to Not interested outcome |
| Murphy, 2020 (46) | Review study |
| Pazzi, 2020 (47) | Due to Not interested outcome |
| Poedjomartono, 2020 (48) | Due to Not interested outcome |
| Rizal, 2020 (49) | Duplicate |
| Sargowo, 2020 (50) | Due to insufficient data |
| Chan, 2021 (51) | Review study |
| Deng, 2021 (52) | Due to Not interested outcome |
| Ghalavand, 2021 (53) | Due to Not interested study design |
| Liemena, 2021 (54) | Abstract |
| Levy, 2021 (55) | Due to Not interested outcome |
| Pazzi, 2021 (56) | Due to Not interested study design |
| Rašeta, 2021 (57) | Due to Not interested study design |
| Ren, 2021 (58) | Animal study |
| Wu, 2021 (59) | Due to Not interested outcome |
| Adrian, 2022 (60) | Abstract |
| Babamiri, 2022 (61) | Duplicate |
| Chen, 2023 (62) | Duplicate |
| Wang, 2023 (63) | Animal study |
| Wu, 2024 (64) | Study protocol |

**Supplementary Table 3.** Summary of ongoing clinical trials investigating the effects of Ganoderma supplementation on health-related indices, providing insights into future research directions and potential advancements in the field

| **Disease** | **Ganoderma composition** | **Clinical trials number** | **phase** |
| --- | --- | --- | --- |
| Osteosarcoma | Spore powder | NCT04319874 | II |
| Physical fitness | Cordyceps sinensis | NCT01718548 | Unknown |
| Gastrointestinal neoplasms | Spore lipids | NCT02785523 | III |
| Head and neck cancer | Spore powder | NCT02238587 | Unknown |
| Prostate cancer | Mikei red reishi essence EX | NCT03589781 | Unknown |
| Eczema | Detox tea | NCT02533635 | I/II |
| Parkinson | Ganoderma Lucidum | NCT03594656 | III |
| idiopathic uveitis | Beta-1,3/1,6-D-Glucan | NCT04162314 | II/III |
| Ulcerative Colitis | Beta-1,3/1,6-D-Glucan | NCT04029649 | II/III |
| Cardiometabolic Syndrome | β-1,3/1,6-D-Glucan | NCT05079529 | II |
| non-small cell lung cancer | spore powder | NCT02844114 | 0 |

**(a)**

**(b)**

**(c)**

**(d)**

**(e)**

**Supplementary Figure 1.**  Forest plot of the effects of Ganoderma supplementation on anthropometric measures (a: body mass index, b: body fat percentage, c: waist circumference, d: weight, e: waist-to-hip ratio)

**(a)**

**(b)**

**Supplementary Figure 2.** Forest plot of the effects of Ganoderma supplementation on blood pressure (a: diastolic blood pressure, b: systolic blood pressure)

**(a)**

**Supplementary Figure 3.** Forest plot of the effects of Ganoderma supplementation on glycemic profile (a: fasting glucose)

**(a)**

**(b)**

**Supplementary Figure 4.** Forest plot of the effects of Ganoderma supplementation on inflammatory markers (a: C-reactive protein, b: TNF-α)

**(a)**

**(b)**

**(c)**

**(d)**

**Supplementary Figure 5.** Forest plot of the effects of Ganoderma supplementation on lipid profile (a: high-density lipoprotein, b: low-density lipoprotein, c: total cholesterol, d: triglycerides)

**(a)**

**(b)**

**(c)**

**Supplementary Figure 6.** Forest plot of the effects of Ganoderma supplementation on liver function tests (a: alanine aminotransferase, b: aspartate aminotransferase, c: creatinine)

**(a)**

**(b)**

**(c)**

**(d)**

**Supplementary Figure 7.** Forest plot of the effects of Ganoderma supplementation on oxidative stress parameters (a: ferric reducing ability of plasma, b: glutathione peroxidase, c: malondialdehyde, d: superoxide dismutase)

**Supplementary Figure 8.** Forest plot of the effects of Ganoderma supplementation on heart rate

**
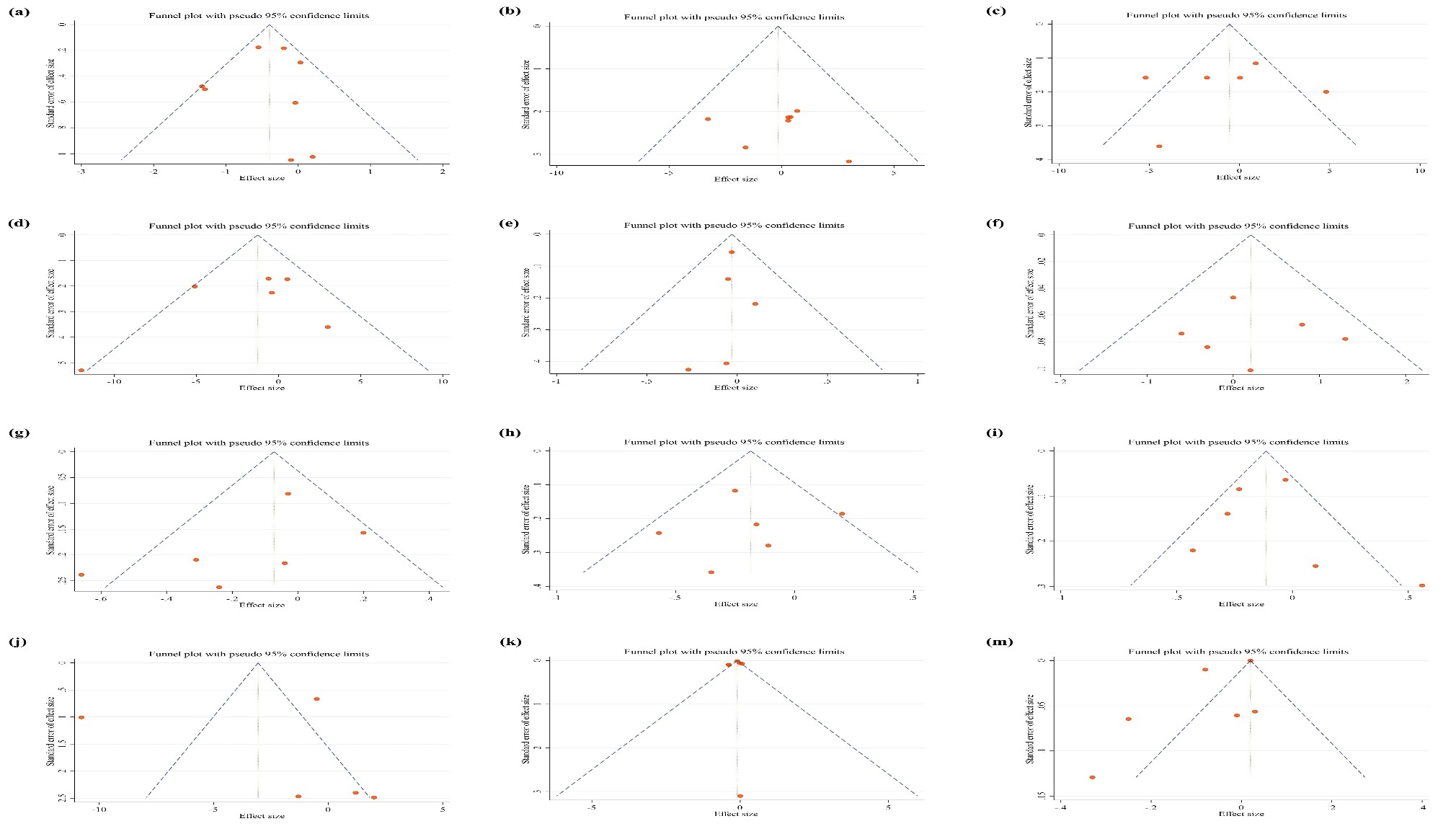
**

**Supplementary Figure 9.** Funnel plot assessing potential publication bias in the meta-analysis of Ganoderma supplementation on health-related indices (a: body mass index, b: weight, c: diastolic blood pressure, d: systolic blood pressure, e: fasting glucose, f: high-density lipoprotein, g: low-density lipoprotein, h: total cholesterol, i: triglycerides, j: alanine aminotransferase, k: creatinine, m: malondialdehyde)

# **Supplemental** **References**

1. Soo T. Effective dosage of the extract of Ganoderma lucidum in the treatment of various ailments. 1996.

2. Gao Y, Zhou S, Chen G, Dai X, Ye J. A phase I/II study of a Ganoderma lucidum (Curt.: Fr.) P. Karst. Extract (Ganopofy) in patients with advanced cancer. International Journal of Medicinal Mushrooms. 2002;4(3).

3. Gao Y, Dai X, Chen G, Ye J, Zhou S. A randomized, placebo-controlled, multicenter study of Ganoderma lucidum (W. Curt.: Fr.) Lloyd (Aphyllophoromycetideae) polysaccharides (Ganopoly®) in patients with advanced lung cancer. International Journal of Medicinal Mushrooms. 2003;5(4).

4. Gao Y, Zhou S, Jiang W, Huang M, Dai X. Effects of Ganopoly®(A ganoderma lucidum polysaccharide extract) on the immune functions in Advanced‐Stage cancer patients. Immunological investigations. 2003;32(3):201-15.

5. Gao Y, Lan J, Dai X, Ye J, Zhou S. A phase I/II study of Ling Zhi mushroom Ganoderma lucidum (W. Curt.: Fr.) Lloyd (Aphyllophoromycetideae) extract in patients with type II diabetes mellitus. International Journal of Medicinal Mushrooms. 2004;6(1).

6. Wachtel-Galor S, Tomlinson B, Benzie IF. Ganoderma lucidum (‘Lingzhi’), a Chinese medicinal mushroom: biomarker responses in a controlled human supplementation study. British Journal of Nutrition. 2004;91(2):263-9.

7. Zhang H-N, Lin ZB. Hypoglycemic effect of Ganoderma lucidum polysaccharides. Acta Pharmacologica Sinica. 2004;25(2):191-5.

8. Gao Y, Tang W, Dai X, Gao H, Chen G, Ye J, et al. Effects of water-soluble Ganoderma lucidum polysaccharides on the immune functions of patients with advanced lung cancer. Journal of medicinal food. 2005;8(2):159-68.

9. Kwok Y, Ng KF, Li CC, Lam CC, Man RY. A prospective, randomized, double-blind, placebo-controlled study of the platelet and global hemostatic effects of Ganoderma lucidum (Ling-Zhi) in healthy volunteers. Anesthesia & Analgesia. 2005;101(2):423-6.

10. Tang W, Gao Y, Chen G, Gao H, Dai X, Ye J, et al. A randomized, double-blind and placebo-controlled study of a Ganoderma lucidum polysaccharide extract in neurasthenia. Journal of medicinal food. 2005;8(1):53-8.

11. Xi Bao Y, Kwok Wong C, Kwok Ming Li E, Shan Tam L, Chung Leung P, Bing Yin Y, et al. Immunomodulatory effects of lingzhi and san-miao-san supplementation on patients with rheumatoid arthritis. Immunopharmacology and immunotoxicology. 2006;28(2):197-200.

12. Hijikata Y, Yamada S, Yasuhara A. Herbal mixtures containing the mushroom Ganoderma lucidum improve recovery time in patients with herpes genitalis and labialis. The Journal of Alternative and Complementary Medicine. 2007;13(9):985-7.

13. Wicks SM, Tong R, Wang C-Z, O'Connor M, Karrison T, Li S, et al. Safety and tolerability of Ganoderma lucidum in healthy subjects: a double-blind randomized placebo-controlled trial. The American Journal of Chinese Medicine. 2007;35(03):407-14.

14. Noguchi M, Kakuma T, Tomiyasu K, Kurita Y, Kukihara H, Konishi F, et al. Effect of an extract of Ganoderma lucidum in men with lower urinary tract symptoms: a double‐blind, placebo‐controlled randomized and dose‐ranging study. Asian journal of andrology. 2008;10(4):651-8.

15. Noguchi M, Kakuma T, Tomiyasu K, Yamada A, Itoh K, Konishi F, et al. Randomized clinical trial of an ethanol extract of Ganoderma lucidum in men with lower urinary tract symptoms. Asian journal of andrology. 2008;10(5):777-85.

16. Wang C, Tschen J, Sheu W. Ganoderma lucidum on metabolic control in type 2 diabetes subjects: A double-blinded placebo control study. Journal of Internal Medicine Taiwan. 2008;19:15-60.

17. Zhuang SR, Chen SL, Tsai JH, Huang CC, Wu TC, Liu WS, et al. Effect of citronellol and the Chinese medical herb complex on cellular immunity of cancer patients receiving chemotherapy/radiotherapy. Phytotherapy Research: An International Journal Devoted to Pharmacological and Toxicological Evaluation of Natural Product Derivatives. 2009;23(6):785-90.

18. Oka S, Tanaka S, Yoshida S, Hiyama T, Ueno Y, Ito M. Ganoderma lucidum mycelia suppresses the development of colorectal adenomas. Hiroshima J Med Sci. 2010;59(1):1-6.

19. Rubel R, Dalla Santa HS, Fernandes LC, Bonatto SJ, Bello S, Figueiredo BC, et al. Hypolipidemic and antioxidant properties of Ganoderma lucidum (Leyss: Fr) Karst used as a dietary supplement. World Journal of Microbiology and Biotechnology. 2011;27:1083-9.

20. Chu TT, Benzie IF, Lam CW, Fok BS, Lee KK, Tomlinson B. Study of potential cardioprotective effects of Ganoderma lucidum (Lingzhi): results of a controlled human intervention trial. British Journal of Nutrition. 2012;107(7):1017-27.

21. Zhuang S-R, Chiu H, Chen S, Tsai J, Lee M, Lee H, et al. Effects of a Chinese medical herbs complex on cellular immunity and toxicity-related conditions of breast cancer patients. British Journal of Nutrition. 2012;107(5):712-8.

22. Rossi P, Buonocore D, Altobelli E, Brandalise F, Cesaroni V, Iozzi D, et al. Improving training condition assessment in endurance cyclists: effects of Ganoderma lucidum and Ophiocordyceps sinensis dietary supplementation. Evidence‐Based Complementary and Alternative Medicine. 2014;2014(1):979613.

23. Klupp NL, Chang D, Hawke F, Kiat H, Cao H, Grant SJ, et al. Ganoderma lucidum mushroom for the treatment of cardiovascular risk factors. Cochrane Database of Systematic Reviews. 2015(2).

24. Liu C, Dunkin D, Lai J, Song Y, Ceballos C, Benkov K, et al. Anti-inflammatory effects of Ganoderma lucidum triterpenoid in human crohn's disease associated with downregulation of NF-κB signaling. Inflammatory bowel diseases. 2015;21(8):1918-25.

25. Sarker MMR. Antihyperglycemic, insulin-sensitivity and anti-hyperlipidemic potential of Ganoderma lucidum, a dietary mushroom, on alloxan-and glucocorticoid-induced diabetic Long-Evans rats. Functional Foods in Health and Disease. 2015;5(12):450-66.

26. Klupp NL, Kiat H, Bensoussan A, Steiner GZ, Chang DH. A double-blind, randomised, placebo-controlled trial of Ganoderma lucidum for the treatment of cardiovascular risk factors of metabolic syndrome. Scientific reports. 2016;6(1):29540.

27. Sargowo D, Ubaidillah N, Handayani O, Widya A, Vittryaturida V, Siwi K, et al. PS 02-02 Effect Ganoderma lucidum polysaccharide peptides as anti-hypertension, anti-lipid, anti-oxidant, anti-inflammation in high risk patients of atherosclerosis. Journal of Hypertension. 2016;34:e105.

28. Tawasri P, Ampasavate C, Tharatha S, Chiranthanut N, Teekachunhatean S. Effect of oral coadministration of ascorbic acid with ling zhi preparation on pharmacokinetics of ganoderic acid a in healthy male subjects: a randomized crossover study. BioMed Research International. 2016;2016(1):2819862.

29. Wu Y-S, Ho S-Y, Nan F-H, Chen S-N. Ganoderma lucidum beta 1, 3/1, 6 glucan as an immunomodulator in inflammation induced by a high-cholesterol diet. BMC complementary and alternative medicine. 2016;16:1-11.

30. Chiu H-F, Fu H-Y, Lu Y-Y, Han Y-C, Shen Y-C, Venkatakrishnan K, et al. Triterpenoids and polysaccharide peptides-enriched Ganoderma lucidum: a randomized, double-blind placebo-controlled crossover study of its antioxidation and hepatoprotective efficacy in healthy volunteers. Pharmaceutical biology. 2017;55(1):1041-6.

31. Farikh A. PEMBERIAN PEPTIDA POLISAKARIDA (PSP) SUATU SENYAWA AKTIF DARI GANODERMA LUCIDUM (Β-D-GLUCAN) BERPENGARUH TERHADAP DISLIPIDEMIA DAN INFLAMASI PADA PASIEN RESIKO TINGGI PENYAKIT JANTUNG KORONER. Care: Jurnal Ilmiah Ilmu Kesehatan. 2017;5(1):102-11.

32. Putthapiban P, Sukhumthammarat W, Sriphrapradang C. Concealed use of herbal and dietary supplements among Thai patients with type 2 diabetes mellitus. Journal of Diabetes & Metabolic Disorders. 2017;16:1-7.

33. Tsuk S, Lev YH, Rotstein A, Carasso R, Zeev A, Netz Y, et al. Clinical effects of a commercial supplement of Ophiocordyceps sinensis and Ganoderma lucidum on cognitive function of healthy young volunteers. International Journal of Medicinal Mushrooms. 2017;19(8).

34. Ali NAM, Saeed HA, Othman RT. Immunostimulatory and anti-inflammatory effect of Ganoderma lucidum on breast cancer patients. Asian Pacific Journal of Cancer Biology. 2018;3(2):51-7.

35. Jung S, Son H, Hwang CE, Cho KM, Park SW, Kim HJ. Ganoderma lucidum ameliorates non-alcoholic steatosis by upregulating energy metabolizing enzymes in the liver. Journal of clinical medicine. 2018;7(6):152.

36. Henao SLD, Urrego SA, Cano AM, Higuita EA. Randomized clinical trial for the evaluation of immune modulation by yogurt enriched with β-glucans from lingzhi or reishi medicinal mushroom, Ganoderma lucidum (Agaricomycetes), in children from Medellin, Colombia. International Journal of Medicinal Mushrooms. 2018;20(8).

37. Sargowo D, Ovianti N, Susilowati E, Ubaidillah N, Nugraha AW, Proboretno KS, et al. The role of polysaccharide peptide of Ganoderma lucidum as a potent antioxidant against atherosclerosis in high risk and stable angina patients. Indian heart journal. 2018;70(5):608-14.

38. Adrian LH, Fadlan MR, Sargowo D, editors. The Role of beta-1, 3/1, 6-D-Glucan (Polysaccharide Peptide) of Miselia Ganoderma lucidum Extracts To Improve Endothelial and Myocardial Function in Post-Myocardial Infarction Patients: A Double-Blind Randomized Controlled Trial. EUROPEAN HEART JOURNAL SUPPLEMENTS; 2019: OXFORD UNIV PRESS GREAT CLARENDON ST, OXFORD OX2 6DP, ENGLAND.

39. Nugroho FW, Adrian LH, Sargowo D, editors. The Developmental Role of beta-1, 3/1, 6-D-Glucan (Polysaccharide Peptide) of Miselia Ganoderma lucidum Extracts To TNF-alpha Levels And Left Ventricular Mass And Geometry In Post-Myocardial Infarction Patients: A Double-Blind Randomized Controlled Trial. EUROPEAN HEART JOURNAL SUPPLEMENTS; 2019: OXFORD UNIV PRESS GREAT CLARENDON ST, OXFORD OX2 6DP, ENGLAND.

40. Parravano M, Tedeschi M, Manca D, Costanzo E, Di Renzo A, Giorno P, et al. Effects of Macuprev® Supplementation in age-related macular degeneration: A double-blind randomized morpho-functional study along 6 months of follow-up. Advances in Therapy. 2019;36:2493-505.

41. Peltzer K, Pengpid S. The use of herbal medicines among chronic disease patients in Thailand: a cross-sectional survey. Journal of multidisciplinary healthcare. 2019:573-82.

42. Pratama AR, Sargowo D, editors. The Antiinflammatory Effect OF beta-1, 3/1, 6-D-Glucan (Polysaccharide Peptide) Of Misela Ganoderma Lucidum Extract Administration To Improvement Of LV Ejection Fraction And LVMI In Post Myocardial Infarction Patients: A Double-Blind Randomized Controlled Trial. EUROPEAN HEART JOURNAL SUPPLEMENTS; 2019: OXFORD UNIV PRESS GREAT CLARENDON ST, OXFORD OX2 6DP, ENGLAND.

43. Sugita P, Fadlan M, Sargowo D, Rizal A. Abstract P2071: β-1, 3/1, 6-d-glucan Of Indonesian Ganoderma Lucidum Mycelium Extract Reduces Systolic Blood Pressure & Inflammation In Hypertensive Patients. Hypertension. 2019;74(Suppl_1):AP2071-AP.

44. Grammatikopoulou MG, Gkiouras K, Papageorgiou SΤ, Myrogiannis I, Mykoniatis I, Papamitsou T, et al. Dietary factors and supplements influencing prostate-specific antigen (PSA) concentrations in men with prostate cancer and increased cancer risk: An evidence analysis review based on randomized controlled trials. Nutrients. 2020;12(10):2985.

45. Liu J, Mao JJ, Li SQ, Lin H. Preliminary efficacy and safety of reishi & privet formula on quality of life among non–small cell lung cancer patients undergoing chemotherapy: a randomized placebo-controlled trial. Integrative cancer therapies. 2020;19:1534735420944491.

46. Murphy EJ, Rezoagli E, Major I, Rowan NJ, Laffey JG. β-glucan metabolic and immunomodulatory properties and potential for clinical application. Journal of Fungi. 2020;6(4):356.

47. Pazzi F, Adsuar JC, Domínguez-Muñoz FJ, García-Gordillo MA, Gusi N, Collado-Mateo D, editors. Ganoderma lucidum effects on mood and health-related quality of life in women with fibromyalgia. Healthcare; 2020: MDPI.

48. Poedjomartono B, Faisal A, Nurjanah S. Transarterial Chemoembolization in Hepatocellular Carcinoma: A Clinical Efficacy Study of Ganoderma Lucidum Extract Polysaccharide Peptide β-Glucan. Bali Medical Journal. 2020;9(1):31-5.

49. Rizal A, Sandra F, Fadlan MR, Sargowo D. Ganoderma lucidum polysaccharide peptide reduce inflammation and oxidative stress in patient with atrial fibrillation. The Indonesian Biomedical Journal. 2020;12(4):384-9.

50. Sargowo D, Wihastuti TA, Dewi ES, Saputri DN. The Role of β-1, 3/1, 6-D-Glucan Forms of Polysaccharide Peptide (PsP) from Mycelia Ganoderma lucidum Extracts for Lowering Elevated Total Cholesterol Level in Patients with Heart Failure in Ischaemic Heart Disease. Group.8:16.735.

51. Chan SW, Tomlinson B, Chan P, Lam CWK. The beneficial effects of Ganoderma lucidum on cardiovascular and metabolic disease risk. Pharmaceutical biology. 2021;59(1):1159-69.

52. Deng Y, Ma J, Tang D, Zhang Q. Dynamic biomarkers indicate the immunological benefits provided by Ganoderma spore powder in post-operative breast and lung cancer patients. Clinical and Translational Oncology. 2021;23:1481-90.

53. Ghalavand A, Saki H, Nazem F, Khademitab N, Behzadi Nezhad H, Behbodi M, et al. The effect of ganoderma supplementation and selected exercise training on glycemic control in boys with type 1 diabetes. Jundishapur Scientific Medical Journal. 2021;20(4):356-65.

54. Liemena H, Sargowo D, Nugroho F, Fadlan M, Sugita P. Potent protective effects of polysaccharide peptide on vascular endothelium and cardiac remodelling in post myocardial infarction patients: A prospective double-blind randomized controlled trial. European Heart Journal Acute Cardiovascular Care. 2021;10(Supplement_1):zuab020. 135.

55. Levy M, Wu J-r, Shi J-p, Cheng H-j, Qu X-q, Bernstein I, et al. Proof-of-Concept and Feasibility Study to Evaluate the Effect of β-Glucan on Protective Qi Deficiency in Adults. Chinese journal of integrative medicine. 2021;27:666-73.

56. Pazzi F, Adsuar JC, Domínguez-Muñoz FJ, García-Gordillo MÁ, Gusi N, Collado-Mateo D. Effects of Ganoderma lucidum and Ceratonia siliqua on blood glucose, lipid profile, and body composition in women with fibromyalgia. Nutricion hospitalaria. 2021;38(1):139-45.

57. Rašeta M, Popović M, Beara I, Šibul F, Zengin G, Krstić S, et al. Anti‐inflammatory, antioxidant and enzyme inhibition activities in correlation with mycochemical profile of selected indigenous Ganoderma spp. from Balkan region (Serbia). Chemistry & Biodiversity. 2021;18(2):e2000828.

58. Ren F, Chen Q, Meng C, Chen H, Zhou Y, Zhang H, et al. Serum metabonomics revealed the mechanism of Ganoderma amboinense polysaccharides in preventing non-alcoholic fatty liver disease (NAFLD) induced by high-fat diet. Journal of Functional Foods. 2021;82:104496.

59. Wu J-r, Cheng H-j, Shi J-p, Yin W-d, Wang J, Ou X-q, et al. β-glucan improves protective qi status in adults with protective qi deficiency—a randomized, placebo-controlled, and double-blinded trial. Chinese journal of integrative medicine. 2021:1-9.

60. Adrian L, Sargowo D, Sugita P. The potential beneficial effects of polysaccharide peptide on oxidative stress and lipid profile in post myocardial infarction patients: a double-blind, randomised controlled trial. European Heart Journal. 2022;43(Supplement_1):ehab849. 090.

61. Babamiri S, Mojani Qomi MS, Shiehmorteza M. The Efficacy of Ganoderma lucidum in Overweight Individuals: A Randomized Placebo-controlled trial. Mediterranean Journal of Nutrition and Metabolism. 2022;15(2):263-71.

62. Chen S-N, Nan F-H, Liu M-W, Yang M-F, Chang Y-C, Chen S. Evaluation of Immune Modulation by β-1, 3; 1, 6 D-Glucan Derived from Ganoderma lucidum in Healthy Adult Volunteers, A Randomized Controlled Trial. Foods. 2023;12(3):659.

63. Wang Y, Yu F, Zheng X, Li J, Zhang Z, Zhang Q, et al. Balancing adipocyte production and lipid metabolism to treat obesity-induced diabetes with a novel proteoglycan from Ganoderma lucidum. Lipids in Health and Disease. 2023;22(1):120.

64. Wu T-T, Chen Y-Y, Yuan Z-C, Yang G-W, Zhang G-L. Synergy of de-walled Ganoderma Lucidum spore powder (GLSP) on targeted therapy in advanced non-squamous non-small cell lung cancer with epidermal growth factor receptor (EGFR) mutant: protocol for a randomized, double-blind, placebo-controlled study. BMC Complementary Medicine and Therapies. 2024;24(1):125.
